# Inflammatory Eicosanoids Increase Amyloid Precursor Protein Expression via Activation of Multiple Neuronal Receptors

**DOI:** 10.1038/srep18286

**Published:** 2015-12-17

**Authors:** Katie J. Herbst-Robinson, Li Liu, Michael James, Yuemang Yao, Sharon X. Xie, Kurt R. Brunden

**Affiliations:** 1 Department of Pathology and Laboratory Medicine, Center for Neurodegenerative Disease Research, Perelman School of Medicine, University of Pennsylvania, Philadelphia, Pennsylvania 19104-6323, USA; 2 Department of Biostatistics and Epidemiology, Perelman School of Medicine, University of Pennsylvania, Philadelphia, Pennsylvania 19104-6323, USA

## Abstract

Senile plaques comprised of Aβ peptides are a hallmark of Alzheimer’s disease (AD) brain, as are activated glia that release inflammatory molecules, including eicosanoids. Previous studies have demonstrated that amyloid precursor protein (APP) and Aβ levels can be increased through activation of thromboxane A2-prostanoid (TP) receptors on neurons. We demonstrate that TP receptor regulation of APP expression depends on Gα_q_-signaling and conventional protein kinase C isoforms. Importantly, we discovered that Gα_q_-linked prostaglandin E2 and leukotriene D4 receptors also regulate APP expression. Prostaglandin E2 and thromboxane A2, as well as total APP levels, were found to be elevated in the brains of aged 5XFAD transgenic mice harboring Aβ plaques and activated glia, suggesting that increased APP expression resulted from eicosanoid binding to Gα_q_-linked neuronal receptors. Notably, inhibition of eicosanoid synthesis significantly lowered brain APP protein levels in aged 5XFAD mice. These results provide new insights into potential AD therapeutic strategies.

A key pathological feature of the Alzheimer’s disease (AD) brain is the presence of senile plaques comprised of Aβ peptides, which are proteolytically-derived from the amyloid precursor protein (APP)^1^. These plaques are thought to contribute, either directly or indirectly, to the neuronal dysfunction and dementia associated with AD^2^. Other factors that are believed to contribute to AD pathogenesis include intracellular aggregates of hyperphosphorylated tau protein^3^, oxidative stress^4^, and neuroinflammation^5^.

The inflammation observed in AD brain results largely from increased microglial activation in the vicinity of senile plaques^6^,^7^ and a number of glial-derived inflammatory molecules, including cytokines, chemokines and eicosanoids, as well as oxidizing molecules, have been suggested to exacerbate AD neuropathology^5^,^8^. For example, isoprostane F2αIII (iPF2αIII), a lipid oxidation product thought to be elevated in AD brain^9^,^10^, can activate the thromboxane A2 (TXA2)-prostanoid (TP) receptor on neurons with a resulting increase of APP mRNA stability that leads to enhanced APP expression and Aβ production^11^,^12^. Similarly, TXA2 itself may also be increased in AD brain, as this eicosanoid is produced by activated microglia^13^.

The signaling pathways that underlie the conversion of TP receptor activation into increases of APP expression and Aβ production have not been previously explored, and here we demonstrate the involvement of Gα_q_ and conventional PKC isoforms. Importantly, we have discovered that activation of additional eicosanoid receptors, including those that bind prostaglandin E2 (PGE2) and leukotriene D4 (LTD4), also results in increased APP levels in receptor-transfected cells, as well as in primary rat or mouse neurons. As PGE2, TXA2, and LTD4 can be released from microglia^5^,^14^, with the former shown to be elevated in the cerebrospinal fluid of AD patients^15^,^16^, these studies further implicate glial inflammation in the pathogenesis of AD. An assessment of 5XFAD transgenic mice that develop Aβ plaques revealed an age-dependent elevation of PGE2 and TXA2, as well as APP. Importantly, inhibiting eicosanoid synthesis in aged 5XFAD mice led to significant diminutions of total APP levels and of α- and β-secretase processed COOH-terminal fragments of APP. The results of these studies provide important new insights into the regulation of APP in the AD brain.

## Results

### TP Receptor Regulation of APP and Aβ Synthesis is Dependent on Gα_q_ and Conventional PKC Isoforms

**Figure 1:**
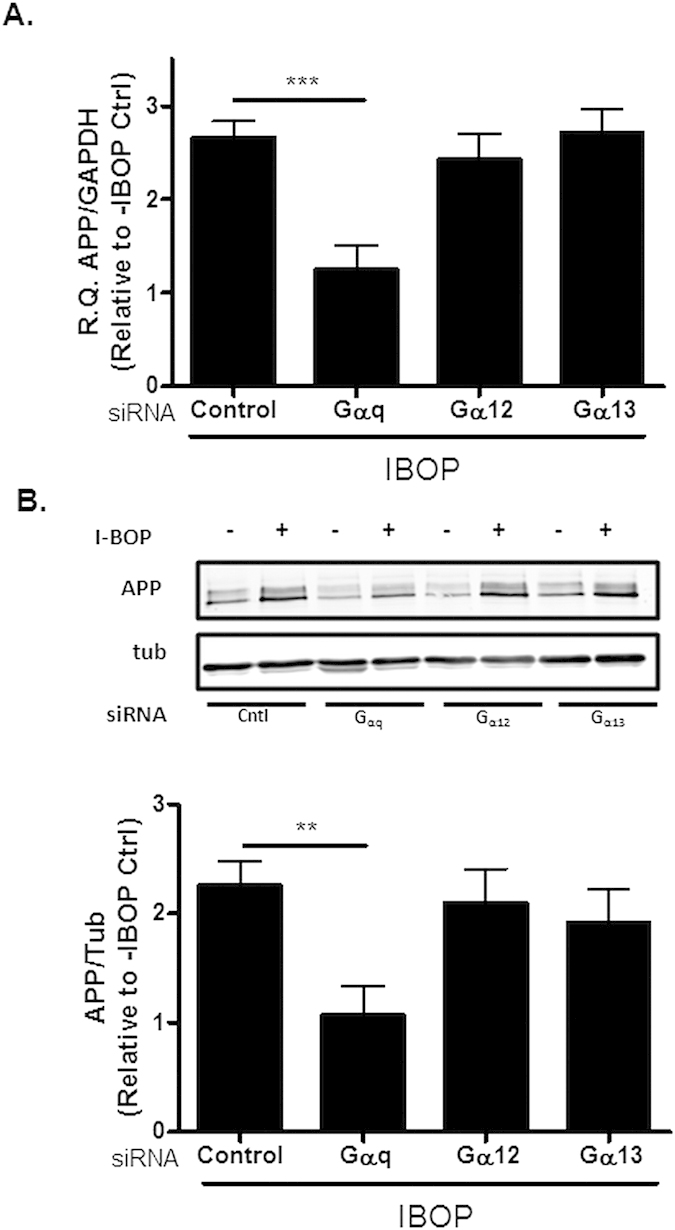
Knockdown of Gα_q_ inhibits TP receptor-mediated increases in APP and Aβ. hTP-hAPP cells were transfected with 50 nM of control siRNA or siRNA directed to Gα_q_, Gα_12_, or Gα_13_ and cultured for 72 h, with IBOP (10 nM) added over the last 48 h. (**A**) qRT-PCR analysis revealed a reduction in IBOP-induced APP mRNA levels only in cells treated with Gα_q_ siRNA [R.Q. (relative quantification) values of APP/GAPDH from qPCR are plotted relative to non-IBOP-treated cells without siRNA addition]. (**B**) Only cells treated with Gα_q_ siRNA showed reduced APP protein expression, as determined by immunoblot analysis with 5685 antibody [values are relative to non-IBOP-treated cells without siRNA addition, with normalization to α-tubulin]. Statistical analyses consisted of a mixed-effects model, with values representing estimates from the least squares means fit of the mixed procedure from 2–6 independent studies with 1–3 replicates for each treatment/study. Error bars represent SEM; **p < 0.01; ***p < 0.001.

To investigate the intracellular signaling molecules involved in the previously reported TP receptor-induced increases in APP expression and Aβ production that result from APP mRNA stabilization^11^,^12^, we utilized siRNA directed to Gα_q_, Gα_12_, and Gα_13_, the G-protein α-subunits most commonly implicated in TP receptor signal transduction^17^. QBI293 cells stably expressing both the human TP receptor (hTP) and human APP_695_ (hTP-hAPP cells) were transfected with control siRNA or siRNA directed to each of the three G-proteins and incubated for 24 h, followed by 48 h treatment in the presence or absence of the TP receptor agonist, [1S-1α,2ß(5Z),3α(1E,3R*),4α)]-7-[-3-(3-hydroxy-4-(4”-iodophenoxy)-1-butenyl)-7-oxabicyclo-[2.2.1]heptan-2-yl]-5-heptenoic acid (IBOP). In agreement with prior studies^11^,^12^, IBOP caused a 2-3-fold increase in APP mRNA and protein in cells receiving control siRNA relative to non-IBOP treated cells (Fig. 1). A significant and nearly complete reduction of the IBOP-induced APP mRNA (Fig. 1A) and protein (Fig. 1B) was observed in cells transfected with siRNA directed to Gα_q_, and not in those that received Gα_12_ or Gα_13_ siRNA. There was a substantial knockdown of each of the Gα mRNAs and corresponding proteins under these conditions (Supplementary Table 1).

Gα_q_ involvement in promoting TP receptor-mediated increases in APP expression was further investigated by pre-incubation of IBOP-treated hTP-hAPP cells with the pan protein kinase C (PKC) inhibitor, Gο6983^18^, to inhibit the activation of PKC downstream of Gα_q_. Cells were also treated with the Rho-associated protein kinase inhibitor, Y-27632^19^, which blocks signaling downstream of Gα_12/13_ activation^20^. Only cells that were pretreated with Gο6983 showed decreased APP mRNA, APP protein, and Aβ1-40 (Supplementary Figure 1) following IBOP stimulation, whereas Y-27632 alone had no effect and its addition did not further increase the effect of Go6983. Although small amounts of Aβ1-42 could be detected in the cellular medium, quantification was not reliable. In conclusion, these studies demonstrate that TP receptor activation of Gα_q_ results in increased levels of APP mRNA and protein, as well as increased production of Aβ.

**Figure 2:**
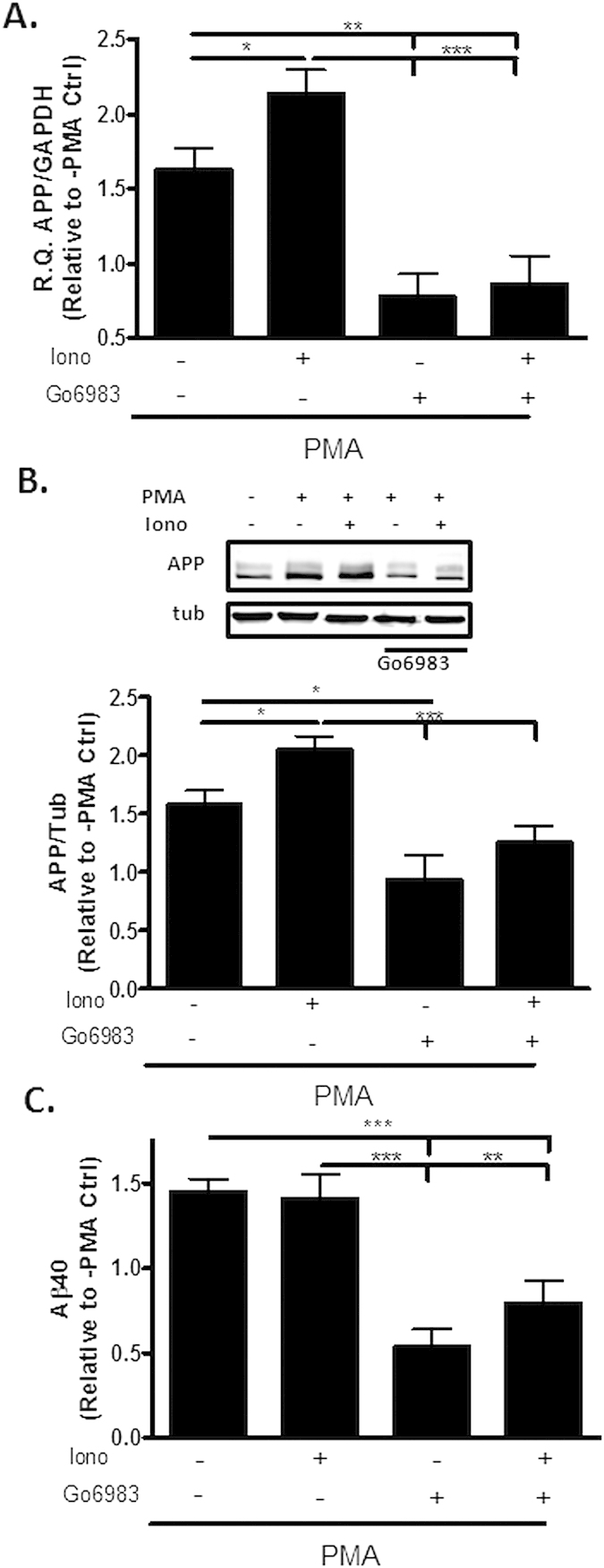
Phorbol ester and ionomycin treatment trigger increased APP expression. Treatment of QBI293 cells stably-expressing hAPP with PMA (1 μM) for 36 h resulted in increased (**A**) APP mRNA [R.Q. values of APP/GAPDH are relative to vehicle-treated cells], (**B**) APP protein expression [(top) representative blot and (bottom) quantification of APP detected with 5685 antibody, with values relative to vehicle-treated cells and normalization to α-tubulin], and (**C**) Aβ1-40 production [values are relative to vehicle-treated cells and normalized to total cellular protein content]. Addition of ionomycin (1 μM) to PMA further increased APP mRNA and protein expression, and Go6983 treatment inhibited these increases. Statistical analyses consisted of a mixed-effects model, with values representing estimates from the least squares means fit of the mixed procedure from 2–4 independent studies with 2–5 replicates for each treatment/study. Error bars represent SEM; *p < 0.05; **p < 0.01; ***p < 0.001.

As the addition of Go6983 revealed that one or more PKC species appeared to be key mediators of TP receptor regulation of APP expression, studies were conducted to examine which specific isoform(s) of PKC are critical to this pathway. There are three broad classes of PKCs^21^: conventional cPKC isoforms that are activated by Ca^++^ and diacylglycerol (DAG); novel nPKC isoforms that are activated by DAG, but are Ca^++^-insensitive; and atypical PKC isoforms that are insensitive to both DAG and Ca^++^, and are activated by other phospholipids. To determine which of the PKC classes are involved in the regulation of APP expression, hTP-hAPP cells were treated with the DAG mimic, phorbol 12-myristate 13-acetate (PMA)^22^, either alone or with the calcium ionophore, ionomycin (Iono)^23^. A significant increase in APP mRNA (Fig. 2A) and protein (Fig. 2B), as well as in Aβ1-40 production (Fig. 2C), were observed upon PMA treatment. The co-treatment of cells with PMA plus ionomycin induced a greater increase of APP mRNA and APP protein expression than was observed with PMA alone (Fig. 2A,B), and the effect of PMA alone or PMA plus ionomycin was blocked by Go6983. A similar trend was observed when Aβ1-40 levels were monitored, although the ionomycin enhancement of the PMA effect was not clearly evident (Fig. 2C). The enhanced APP expression observed with the Ca^++^ ionophore in the presence of PMA suggests that it is primarily cPKC isoforms that are responsible for the effects on APP.

To further define the cPKC isoforms involved in the regulation of APP expression, select cPKC (α and βII) and nPKC (ε) isoforms were knocked down via siRNA in hTP-hAPP cells. Efficient knockdown of the PKC isoforms was observed 72 h after transfection (see Supplementary Table 2), and siRNA-treated cells were incubated in the presence of IBOP. Cells that were treated with siRNA directed against PKCε showed no decrease in APP protein expression (Supplementary Figure 2) relative to cells receiving control siRNA. Conversely, transfection with siRNA directed against PKCα or PKCβII reduced the IBOP-induced increases in APP mRNA and protein, with PKCβ causing a greater reduction. Simultaneous PKCα and PKCβII siRNA addition did not completely inhibit IBOP-induced increases in APP expression, perhaps due to the residual PKCα and PKCβII that remain after knockdown, or because an additional cPKC isoform also contributes partially to the regulation of APP expression. Nonetheless, these findings further confirm that cPKCs are largely responsible for the effects of TP receptor activation on APP expression.

### Additional Eicosanoid Receptors Regulate APP Expression

**Figure 3:**
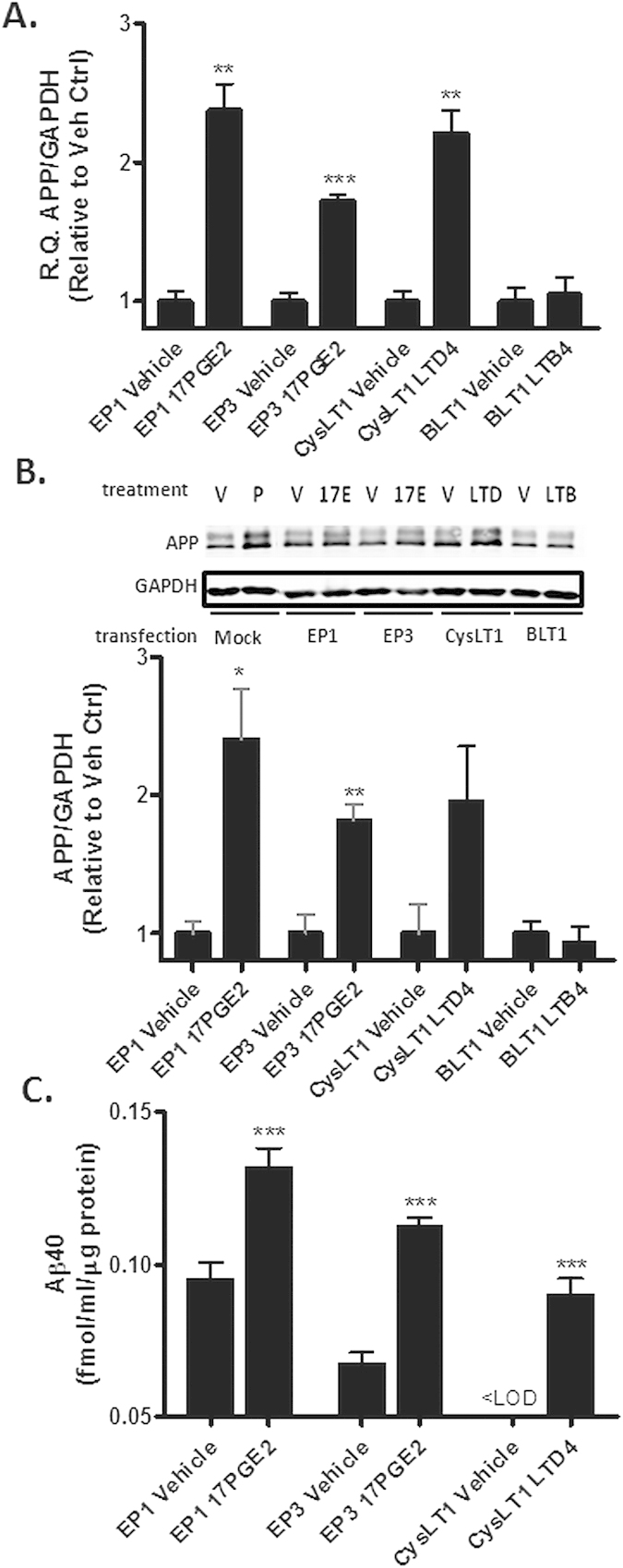
Additional Gα_q_-linked receptors regulate APP expression and Aβ release. hAPP-expressing QBI293 cells were transiently transfected with cDNA encoding the human EP1, EP3, CysLT1 or BLT1 receptors. (**A-C**) The cells were treated for 36 h with vehicle or with agonist (100 nM 17PGE2 for EP1 and EP3; 1 μM LTD4 for CysLT1; 1 μM LTB4 for BLT1), followed by analysis of (**A**) APP mRNA [R.Q. values of APP/GAPDH are relative to vehicle-treated cells], (**B**) APP protein [(top) representative blot and (bottom) quantification of APP with 5685 antibody relative to the vehicle-treated cells, normalized to GAPDH], or (**C**) Aβ1-40 released into the culture medium. The presented data are the means obtained from a single independent study conducted with each treatment in triplicate. For Aβ1-40 ELISA determinations, each sample was analyzed in triplicate. A second independent study was conducted to confirm the findings (not shown). A two-tailed, t-test was applied to test if the values of the treatment groups differed relative to receptor-transfected cells in the absence of agonist. A one sample t-test was conducted in (**C**) for the CysLT1 samples, as the Aβ1-40 was below the level of detection in the vehicle group. Error bars represent SEM; *p < 0.05; **p < 0.01; ***p < 0.001. <LOD = below limit of detection.

The aforementioned findings raised the question of whether other Gα_q_-coupled receptors, in addition to the TP receptor, might also affect APP levels. In particular, the compelling evidence of a connection between neuroinflammation and AD pathology prompted an investigation of additional Gα_q_-linked receptors that, like the TP receptor, are reported to be expressed on neurons and can be activated by inflammatory eicosanoids. This led to an evaluation of the PGE2 receptors, EP1 and EP3^24^, the LTD4 receptor, CysLT1^25^, and the LTB4 receptor, BLT1^26^. QBI293 cells stably-expressing hAPP were transfected with constructs encoding each of these receptors, and these cells were subsequently exposed to known agonists. Ligand activation of the EP1, EP3, and CysLT1 receptors, but not the BLT1 receptor, led to increases in APP mRNA (Fig. 3A) and protein (Fig. 3B), as well as Aβ(1–40) (Fig. 3C). In the case of the EP1 and EP3 receptor-expressing cells, some Aβ(1–40) was produced in the absence of agonist, presumably due to a constitutive receptor activity. The lack of APP increase after BLT1 transfection was not the result of poor receptor expression, as qPCR analysis revealed the presence of appreciable transcript (Supplementary Table 3). Thus, activation of multiple Gα_q_-linked eicosanoid receptors results in enhanced APP expression and Aβ release. However, this does not appear to be a feature of all Gα_q_-coupled receptors, as activation of the BLT1 receptor or the unrelated Gα_q_-linked angiotensin I receptor (not shown) did not result in changes of APP expression.

**Figure 4:**
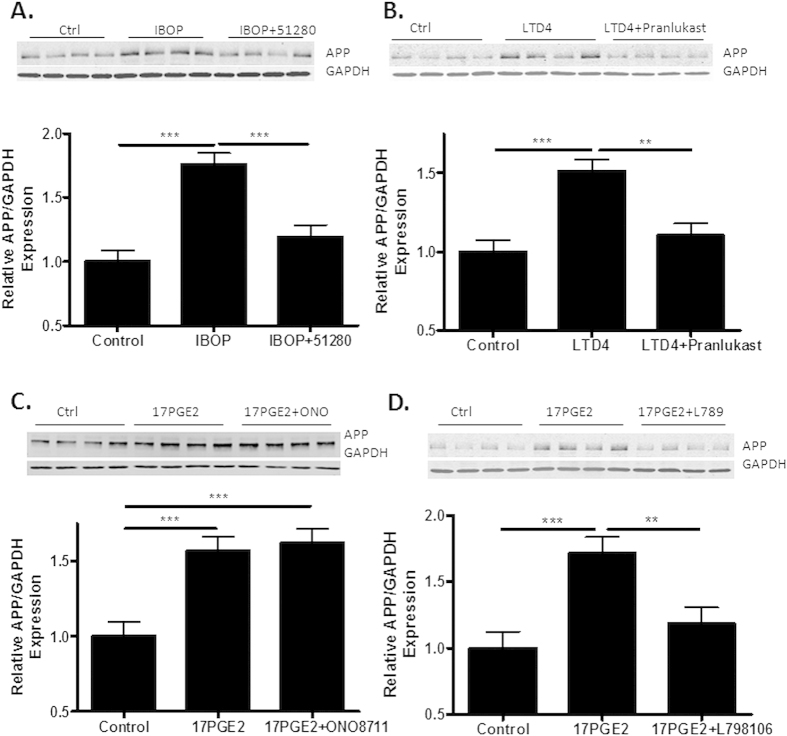
Activation of eicosanoid receptors increases APP expression in primary rat hippocampal neurons. Rat hippocampal neurons that were grown in culture for 14 days were treated for 2 days with receptor agonist alone or in combination with a specific receptor antagonist, and APP expression was compared to that of control cultures by immunoblotting (22C11 antibody). (**A**) IBOP (100 nM) increased APP expression and was blocked by the TP receptor antagonist, CNDR-51280 (10 μM); (**B**) LTD4 (1 μM) increased APP expression and was inhibited by the CysLT1 receptor antagonist, Pranlukast (1 μM); and (**C,D**) 17PGE2 (1 μM) enhanced APP expression, and was not inhibited by the EP1 receptor antagonist, ONO8711 (20 nM), but was blocked by the EP3 receptor antagonist, L798,106 (30 nM). Statistical analyses consisted of a mixed-effects model, with values representing estimates from the least squares means fit of the mixed procedure from 3 independent studies, with 4 replicates per study. Error bars represent SEM; **p < 0.01; ***p < 0.001.

Each of the inflammatory eiscosanoids that were found to increase APP expression were examined for their effects on primary rat hippocampal neuron cultures that were treated with an anti-mitotic (cytosine arabinoside) to deplete dividing, non-neuronal cells. Consistent with prior observations^11^, IBOP addition resulted in a significant increase of APP (Fig. 4A) that could be blocked with the specific antagonist, CNDR-51280 [compound 5 in^12^]. Treatment of the neurons with LTD4 or the PGE2 analogue, 17PGE2, also led to increased APP expression (Fig. 4B–D). The LTD4-mediated increase of APP was effectively inhibited with the CysLT1 receptor antagonist, pranlukast^27^ (Fig. 4B). Interestingly, the EP1 antagonist, ONO8711^28^, did not block the 17PGE2-induced elevation of APP (Fig. 4C), whereas the EP3 antagonist, L798,106^29^, significantly inhibited the effect of 17PGE2 on APP expression (Fig. 4D). These data thus imply that the EP3 receptor, but not the EP1 receptor, mediated the 17PGE2-triggered increase of APP expression in rat hippocampal neuron cultures.

**Figure 5:**
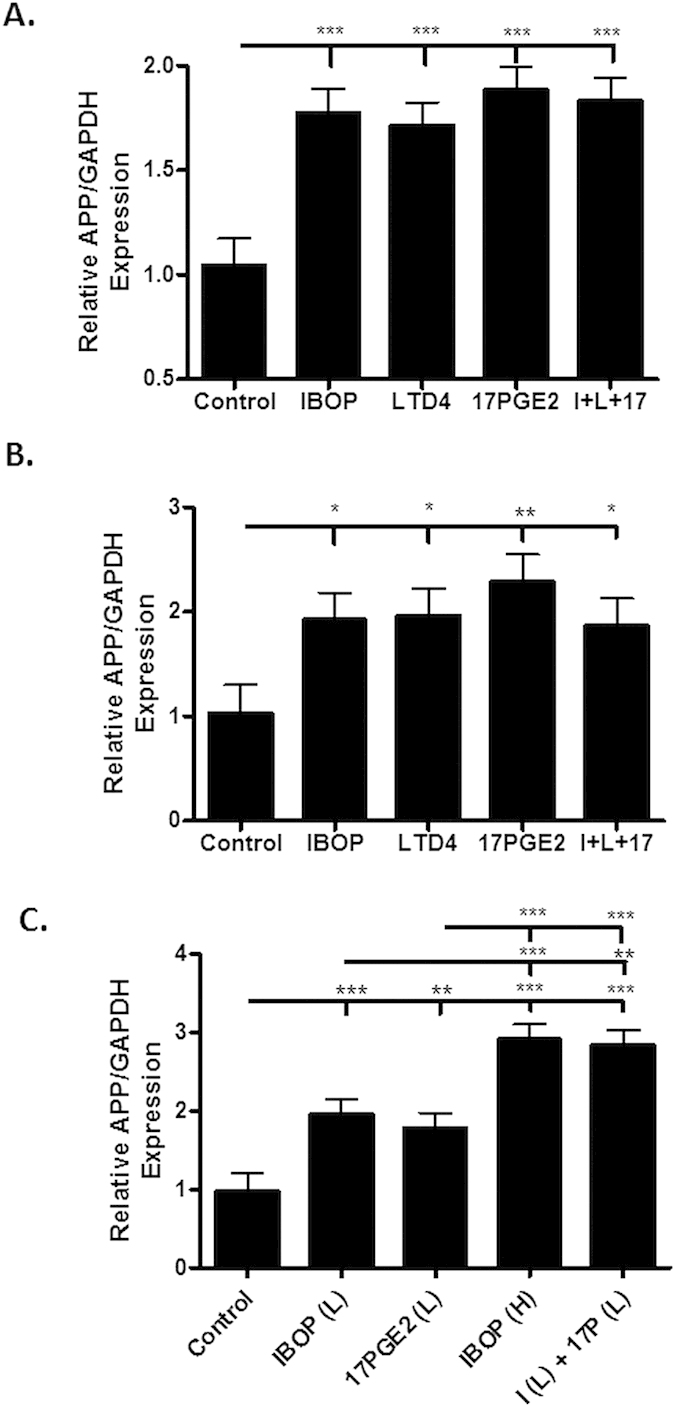
Activation of eicosanoid receptors in primary neurons with saturating and sub-saturating concentrations of receptor agonists. Rat hippocampal neurons (**A**) or mouse cortical neurons (**B**) that were grown in culture for 14 days were treated for 2 days with vehicle or high concentrations of IBOP (100 nM), LTD4 (1 μM), 17PGE2 (1 μM), or the combination of these agonists (I + L + 17). In (**C**), rat hippocampal neurons were treated with vehicle or sub-saturating concentrations of IBOP (50 nM; IBOP(L)), 17PGE2 (0.5 μM; 17PGE2(L)), or the combination of these agonists (I(L) + 17(L)). In addition, neurons were also treated with a high concentration of IBOP (100 nM; IBOP(H)). APP was quantified from immunoblots (22C11 antibody), and statistical analyses consisted of a mixed-effects model, with values representing estimates from the least squares means fit of the mixed procedure from 3–4 independent studies, with 2–3 replicates per study. Error bars represent SEM; *p<0.05, **p < 0.01; ***p < 0.001.

The relative magnitude of the increase of APP expression in the rat neurons was similar upon activation of each of the eicosanoid receptors, suggesting a similar degree of intracellular signaling via cPKC isoforms. To examine this further, additional studies were conducted in which high concentrations of the receptor agonists were added singly, or concurrently, to rat hippocampal neuron cultures. The simultaneous addition of all three stimulatory eicosanoids did not increase APP expression beyond that observed when each of these agents was added individually (Fig. 5A). A similar observation was made with primary mouse cortical neuron cultures (Fig. 5B). Although the addition of saturating levels of multiple agonists to the eicosanoid receptors did not lead to additive increases of APP expression, concurrent treatment of rat hippocampal neurons with sub-saturating concentrations of IBOP and 17PGE2 resulted in an increase of APP expression relative to that obtained with each agonist added alone (Fig. 5C).

### Evidence of Eicosanoid Enhancement of APP Expression in the 5XFAD Transgenic Mouse Model of AD

**Figure 6:**
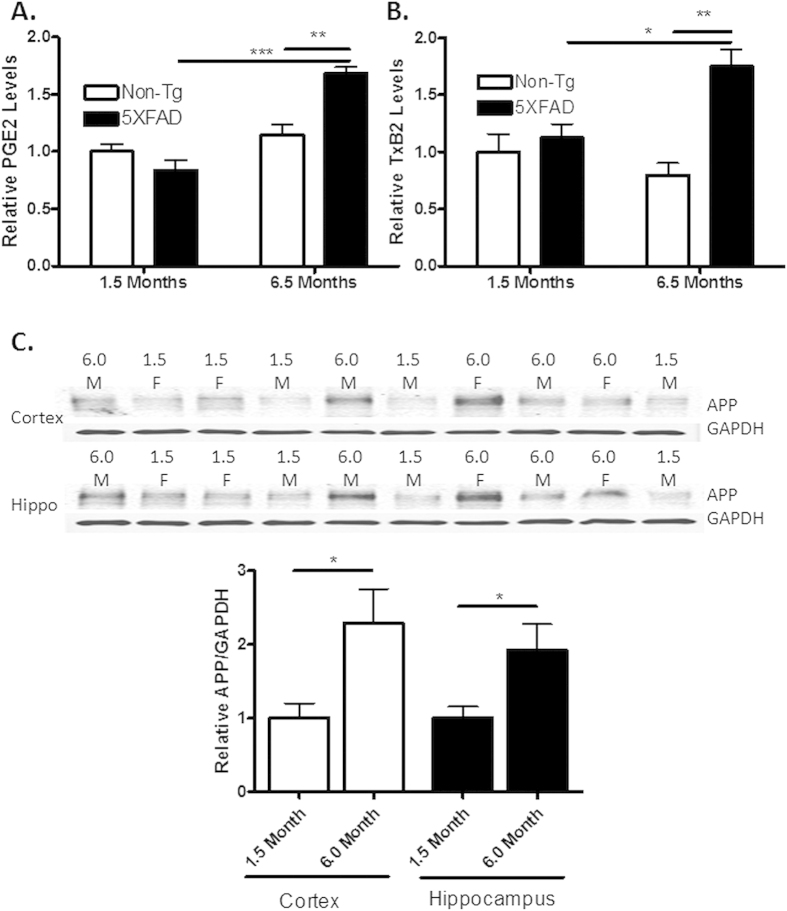
Aged 5XFAD transgenic mice show increased levels of PGE2 and TXB2, as well as APP. Whole hemisphere brain homogenates from 5XFAD mice and non-transgenic (non-Tg) littermates that were 1.5 or 6.5 months of age were analyzed by LC-MS/MS for (**A**) PGE2 and (**B**) TXB2 (n = 3–4 per group). In (**C**), the level of total APP expression in cortical and hippocampal brain homogenates from 1.5 month old and 6.0 month old 5XFAD mice was determined by immunoblotting (22C11 antibody), with normalization to GAPDH. Lane loading in the immunoblot was randomized for study blinding during quantification (n = 5 per group; 2 females (F) and 3 males (M) in each age group). Statistical analyses consisted of a one-way ANOVA with a Tukey’s multiple comparison test for (**A**) and (**B**), and a two-tailed t-test in (**C**). Error bars represent SEM; *p < 0.05; **p < 0.01; ***p < 0.001.

**Figure 7:**
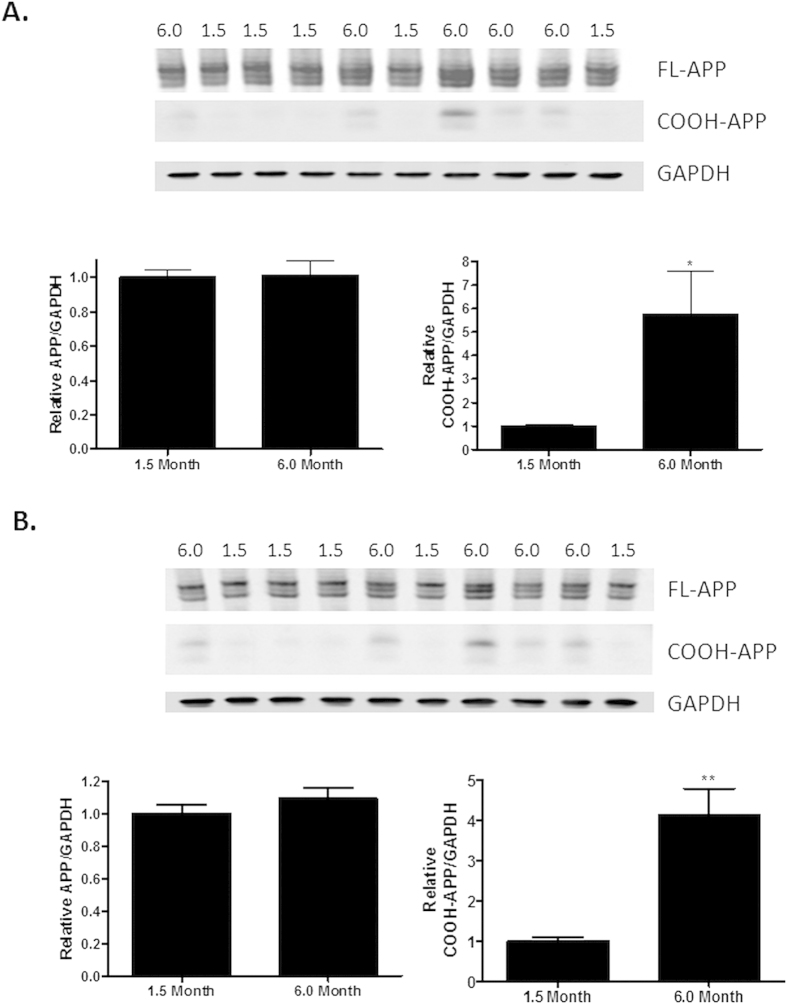
COOH-terminal APP antibody (5685) reveals increased COOH-terminal APP fragments, but not intact APP, in aged 5XFAD mice. APP expression in (**A**) cortical and (**B**) hippocampal brain homogenates from 1.5 month old and 6.0 month old 5XFAD mice (same samples as in Fig. 6) was determined by immunoblotting with the COOH-terminal 5685 APP antibody after protein separation on a 4–12% gradient gel, with normalization to GAPDH. Lane loading in the immunoblot was as in Fig. 6. Statistical analyses consisted of a two-tailed t-test N = 5/group; *p < 0.05; **p < 0.01.

The discovery that multiple eiscosanoids could increase APP expression through interaction with neuronal receptors suggested that a vicious cycle may ensue in the AD brain, whereby initial glial activation by Aβ oligomers and/or plaques results in a release of inflammatory eicsosanoids that subsequently up-regulate APP and Aβ production. PGE2 is reported to be elevated within the CSF of AD patients^16^, and to further explore whether there are increased eicosanoid levels in a mouse model with Aβ plaques, we measured both PGE2 and the TXA2 metabolite, TXB2, in 5XFAD transgenic mice in which robust Aβ deposition and glial inflammation are observed with age^30^. The levels of PGE2 (Fig. 6A) and TXB2 (Fig. 6B) were comparable in brain homogenates from 1.5-month old 5XFAD transgenic mice and age-matched non-transgenic littermates, an age where the 5XFAD mice show relatively little Aβ deposition^30^. In contrast, significant increases in these eicosanoids were observed in the brains of 6.5-month old 5XFAD mice, which have profound Aβ deposition^30^, relative to age-matched non-transgenic mice and to the younger 5XFAD mice (Fig. 6A,B). Attempts were made to quantify LTD4 levels in 5XFAD mouse brain homogenates, but the amounts were below the level of detection by LC-MS/MS. The observation of an age-dependent increase of brain eicosanoids in 5XFAD mice suggested that there may be a comparable increase of APP expression in the older mice due to activation of neuronal receptors, as described above. Notably, total APP levels in the cortex and hippocampus were elevated approximately two-fold in 6-month old 5XFAD mice relative to younger mice (Fig. 6C and Supplementary Figure 3). The age-dependent increase in APP appeared to occur in both genders of 5XFAD mice, although the inclusion of only two female mice in both the 1.5- and 6-month old groups did not allow for a statistical comparison of age effect by gender. Interestingly, whereas a significant increase in APP levels was observed with antibodies that recognize α- and β-secretase cleaved APP (i.e., sAPP) as well as intact APP (Fig. 6C; 22C11 antibody and Supplementary Figure 3B; Karen antibody), the age-dependent elevation in APP was not observed when only intact APP was measured with a COOH-terminal antibody (Fig. 7 and Supplementary Figure 4; 5685 antibody). This suggested that there was an increase of sAPP in the older transgenic mice, and analysis of APP COOH-terminal fragments generated after α- and β-secretase cleavage of APP also revealed a significant increase in these fragments in the older 5XFAD mice (Fig. 7). Notably, both the larger COOH-terminal fragment, which should correspond to the C99 fragment generated after β-secretase cleavage of APP, and the smaller C83 COOH-terminal fragment that results from α-secretase cleavage of APP^31^, are increased in the older 5XFAD mice. This commensurate increase of sAPP and both α/β-secretase-generated COOH-terminal fragments of APP in the 6.0-month old 5XFAD mice suggests that there is an overall increase of APP expression in the older mice, with the majority of the increased APP undergoing processing, rather than an elevation of sAPP species that results from slowed sAPP degradation or a specific change in activity of one of the enzymes that cleaves APP. A future comparison of APP mRNA levels in young and aged 5XFAD mice would further strengthen this interpretation.

**Figure 8:**
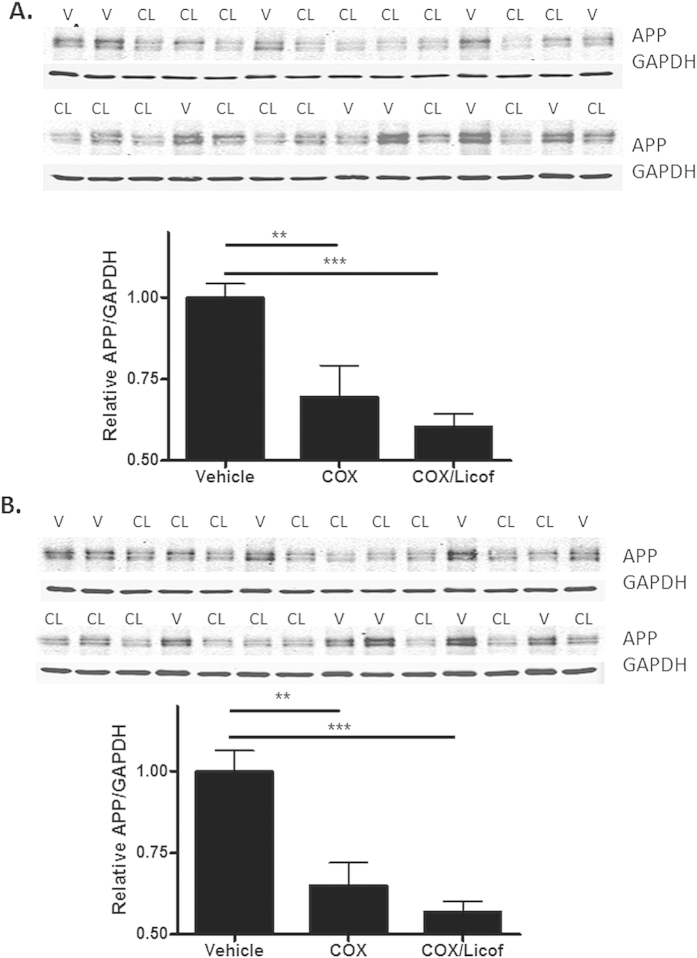
Inhibition of eicosanoid production reduces APP expression in aged 5XFAD transgenic mice. 5XFAD mice (5.5–6.0 months of age) were provided drinking water containing SC-560 and rofecoxib for a total of 7 days (COX group; 3 females and 6 males). Another group of similarly aged 5XFAD mice received the COX inhibitor mixture supplemented with licofelone (COX/Licof group; 5 females and 4 males). A group of age-matched 5XFAD mice received drinking water containing the vehicle only (5 females and 5 males). Quantification of total APP (22C11 antibody) normalized to GAPDH for cortical (**A**) and hippocampal (**B**) homogenates revealed a significant reduction in both the COX and COX/Licof treatment groups compared to the vehicle group. Primary immunoblots are shown for the COX/Licof-treated (CL) and vehicle-treated (V) mice, with lane loading randomized for study blinding during quantification. The 9 COX/Licof inhibitor-treated samples were run on each gel, with the 10 vehicle-treated samples split between the two gels. The relative APP/GAPDH value for each drug-treated mouse sample was normalized to the vehicle mean on each gel, with the final APP/GAPDH value consisting of the average sample value derived from the two separate gels. A similar analysis was conducted for the COX inhibitor treatment group. Statistical analyses consisted of a one-way ANOVA with a Tukey’s multiple comparison test. *p < 0.05; **p < 0.01, ***p<0.001.

To confirm an involvement of eicosanoids in the regulation of APP in aged 5XFAD mice, we inhibited the key enzymes involved in their synthesis. PG and TX synthesis depends on the metabolic conversion of arachidonic acid to PGH2 by COX-1 and/or COX-2^32^, and we evaluated known COX inhibitors for their ability to achieve meaningful brain concentrations upon oral administration. Both the selective COX-1 inhibitor, SC-560^33^ and the COX-2 inhibitor, rofecoxib^34^, demonstrated good brain exposures (brain/plasma drug ratios ≥0.3) after oral dosing. This led to the administration of a mixture of these two inhibitors in drinking water to 5.5-6-month old 5XFAD mice for 7 days, with a separate group of mice receiving vehicle only. The drug-treated group had appreciable levels of the two compounds in their brains (Supplementary Table 4) that resulted in a nearly complete reduction of PGE2 and TXB2 (Supplementary Figure 5). An evaluation of combined sAPP and intact APP species by immunoblotting with the 22C11 antibody revealed that the COX inhibitors caused a significant reduction of APP levels of approximately 30% within the cortex (Fig. 8A) and hippocampus (Fig. 8B).

In addition to the evaluation of COX-derived eicosanoids, a potential role of LTD4 was also investigated through inhibition of 5-LOX, the enzyme responsible for the synthesis of cysLTs^32^. As LTD4 and related LT levels were found to be below the limit of LC-MS/MS detection, and because a commercial LTB4 ELISA gave artifactual readings from brain homogenates, we could not directly quantify inhibition of 5-LOX activity through the measurement of these species. Thus, we chose to investigate the potential use of the dual COX/5-LOX inhibitor, licofelone^35^. Although this compound shows poor blood-brain barrier permeability, preliminary studies showed that 0.6 μM brain concentrations could be achieved by administering a high dose (0.7 mg/ml) via drinking water to mice. Importantly, this dose led to ~40–50% reductions in COX activity as assessed by measurements of PGE2 and TXB2 in the brains of aged 5XFAD mice (Supplementary Figure 6). As licofelone has nearly identical IC_50_ values for the inhibition of COX-1, COX-2 and 5-LOX^35^, a ~40–50% inhibition of 5-LOX should also occur at this dose. Attempts to further increase the licofelone dose to increase 5-LOX inhibition could not be achieved because of compound insolubility. Thus, licofelone was added at 0.7 mg/ml to drinking water containing SC-560 and rofecoxib, which was administered to 5.5-6-month old 5XFAD mice for 7 days. As expected, nearly complete inhibition of TXB2 and PGE2 production was observed in the treated mice (Supplementary Figure 5) and 0.6 μM licofelone levels were again achieved in the brain (Supplementary Table 4), although the mice that received the mixture containing licofelone consumed less water than the groups receiving vehicle or the COX inhibitors only, presumably due to taste aversion since the mice otherwise seemed unaffected, without a significant change in body weight. This resulted in lower SC-560 and rofecoxib brain concentrations than achieved upon treatment with COX inhibitors only (Supplementary Table 4). The addition of licofelone to the COX inhibitors resulted in a greater suppression of APP expression in both the cortex (40%; Fig. 8A) and hippocampus (43%; Fig. 8B) than obtained with the COX inhibitors alone. However, the difference between the COX-only and COX/Licof treatment groups did not reach statistical significance under these conditions of partial 5-LOX inhibition. Thus, although these data are suggestive, we cannot definitively conclude that the combination of COX and 5-LOX inhibition results in a greater decrease of APP levels than is achieved with COX inhibitors only.

Interestingly, the reduction in total APP upon treatment of the aged 5XFAD mice with inhibitors of eicosanoid synthesis appeared to result from a decrease in sAPP species, as no changes in intact APP were observed when brain samples were analyzed with the COOH-terminal 5685 APP antibody (Supplementary Figure 7). However, there was a significant reduction in COOH-terminal APP fragments in the inhibitor-treated 5XFAD mice, without evidence of a preferential reduction of C99 vs. C83 fragments (Supplementary Figure 7). The coordinate changes in sAPP and COOH-terminal APP fragments are essentially identical to what was observed as the 5XFAD mice aged from 1.5 months to 6.0 months of age. Thus, these data provide further evidence that the increase of APP observed in aged 5XFAD mice results from the age-dependent elevation in eicosanoid levels.

Whereas the reduction of eicosanoids in the aged 5XFAD mice led to a significant diminution of COOH-terminal APP fragments, total Aβ levels in the brains of these mice did not change after the 7 days of drug treatment (Supplementary Figure 8). As nearly all of the Aβ in 5XFAD mice of this age is within insoluble plaques, it may not be surprising that a short-term reduction in APP would not lead to meaningful change of total Aβ. In fact, this result is consistent with what has been observed in APP transgenic mice treated with a potent β-secretase inhibitor^36^, where reduced Aβ production did not lead to a significant reduction of total Aβ after only one week of dosing.

## Discussion

There is value in identifying new mechanisms and targets for regulating Aβ levels in AD brain. In this regard, antagonism of the TP receptor has been suggested as a potential strategy to reduce Aβ production in AD, as activation of this receptor by iPF2αIII or TXA2 results in increased APP mRNA and APP protein expression, as well as elevated Aβ release^11^,^12^. The TP receptor-mediated increase of APP expression results from mRNA stabilization that does not appear to require 5′- or 3′-untranslated sequences, as this effect can be seen in cellular and animal models utilizing APP transgenes lacking these regions^11^. We have further investigated the intracellular pathways responsible for TP receptor-mediated elevation of APP, and reveal the involvement of cPKC isoforms that are activated via Gα_q_-mediated signaling. The observation that the PKC activator, PMA, increased APP and Aβ production contrasts with earlier studies^37^,^38^ showing reduced Aβ release after phorbol ester treatment of APP-expressing cells due to increased α-secretase activity. However, these prior studies examined acute phorbol ester treatment, whereas our studies were performed over a longer treatment period. The data presented here are consistent with a prior report^39^ of 8 h phorbol ester treatment causing increased APP expression, as well as Aβ production.

A key finding from our studies that has important implications for AD is the identification of the EP1, EP3 and CysLT1 receptors as additional Gα_q_-linked GPCRs that can modulate APP and Aβ expression. This discovery adds to a growing body of evidence implicating glial-derived eicosanoids in AD pathology. For example, genetic knockout of the EP1, EP2 or EP3 receptors has been shown to result in a significant reduction of plaque burden in APP transgenic mice^40^,^41^,^42^ via a number of postulated mechanisms. Similarly, knockout and pharmacological inhibition of the PGE2 EP4 receptor has been reported to reduce plaque burden in an APP Tg mouse model^43^, although an opposite effect was observed in another APP Tg model^44^. There is also evidence of APP regulation in glia via activation of EP2 receptors^45^,^46^. Finally, LTD4 injection into mouse brain was shown to increase expression of APP and Aβ^47^, and 5-LOX-derived LTs have also been suggested to contribute to Aβ plaque deposition through an effect on γ-secretase activity^48^,^49^.

The evidence of eicosanoid contribution to Aβ plaque pathology suggests possible therapeutic strategies to mitigate the effects of these molecules in AD. A dampening of microglial activation might result in the diminished release of these species. However, it could prove difficult to selectively decrease the detrimental aspects of activated microglia without also affecting potential beneficial properties (e.g., Aβ phagocytosis). An alternative approach could be antagonism of eicosanoid receptors, but the evidence implicating multiple neuronal eicosanoid receptors as contributing to the regulation of APP expression and Aβ production presents a multi-target pharmacological challenge. Nonetheless, there may be merit in further exploration of this strategy, as selective antagonism of the most detrimental eicosanoid receptor(s) could provide an effective therapeutic strategy.

Perhaps the most straightforward potential therapeutic strategy to prevent the eicosanoid-driven increases of APP and Aβ in AD would be through inhibition of eicosanoid production, an approach that has been explored with varying results. COX inhibitors have been shown to fairly consistently reduce Aβ pathology in several AD mouse models, although negative reports exist^33^,^50^,^51^ and in some instances reduction of plaques may have resulted in whole or part from the ability of certain non-steroidal anti-inflammatory drugs (NSAIDs), such as ibuprofen, to modulate γ-secretase activity^52^,^53^. Moreover, although multiple epidemiological studies suggest that non-steroidal anti-inflammatory drug (NSAID) regimens can reduce the incidence of AD^51^,^54^, NSAIDs have not generally proven effective in AD clinical trials^55^,^56^,^57^. There are possible explanations for this lack of clinical success. For example, several of the trials utilized COX-2-selective agents, and it may be COX-1 that is up-regulated upon glial activation in AD^33^,^51^. In this regard, asymptomatic individuals treated with the dual COX-1/COX-2 inhibitor naproxen showed some evidence of reduced AD onset 2–3 years after completion of the ADAPT trial, whereas those receiving a COX-2 selective agent did not^58^. Moreover, it is possible that COX inhibition alone may lead to shunting of arachidonic acid to the 5-LOX pathway, resulting in increased production of LTs^59^,^60^. This might cause CysLT1 receptor activation and increased APP and Aβ levels, as well as increased Aβ via enhanced γ-secretase cleavage of APP^48^,^49^. Thus, the utilization of a combination of COX and 5-LOX inhibitors, or dual-acting COX/5-LOX inhibitors^61^, may merit consideration for AD. It has been reported that 5-LOX and COX levels are increased in the AD brain^32^,^48^,^62^,^63^, and combined COX/5-LOX inhibitors should reduce the production of PGs, TXs and LTs. Furthermore, whereas NSAIDs can cause gastrointestinal or cardiovascular complications, and were poorly tolerated by a percentage of AD patients^64^, dual COX/5-LOX inhibitors such as licofelone appear to have decreased side-effects when compared to typical NSAIDs^35^,^65^. Thus, there would appear to be merit in evaluating the effects of prolonged inhibition of both COX and 5-LOX enzymes in APP Tg mouse models of plaque formation to test this therapeutic strategy. However, such an approach would result in a systemic reduction of PGs, TXs and LTs, some of which clearly play a beneficial role within the body, so as with all drugs, a benefit-to-risk assessment would be important if such a strategy were pursued.

In summary, our studies further elucidate how inflammatory eicosanoids might contribute to AD pathogenesis, and provide important new information about GPCR regulation of APP expression. The approximately two-fold increase of APP levels that are observed in culture systems upon activation of the multiple Gα_q_-linked eicosanoid receptors described herein is consistent with reports of elevated APP in AD brain^66^,^67^, as well as the increased APP expression that we observed in plaque-bearing 5XFAD mice. These observations suggest that initial Aβ plaque formation and glial activation in the brain results in the initiation of a vicious cycle, whereby glial-derived eicosanoids may further elevate APP and Aβ expression, thereby accelerating additional plaque deposition. Our data showing that inhibition of eicosanoid synthesis decreases total APP levels and reduces COOH-terminal APP fragments in aged 5XFAD mice supports this cascade hypothesis, and provide impetus to further investigate the contribution of these inflammatory molecules and their receptors to Aβ plaque pathology.

## Methods

### Cell culture and transfection

QBI293 cells expressing hTP and/or hAPP^12^ were grown in DMEM cell culture plus 10% FBS and 1% pen/strep at 37 °C with 5% CO_2_. For studies utilizing siRNA, cells in 6-well plates were transfected with 30–50 nM siRNA via Lipofectamine RNAiMax reagent according to the manufacturer’s protocol (Life Technologies; Grand Island, NY) 18–24 h prior to any subsequent treatment. The following siRNAs were used: Cntl, Gαq and PKCβ siRNA (Santa Cruz Biotechnologies, Inc.; Dallas, TX); Gα_12_ and Gα_13_ (Thermo Scientific; Waltham, MA); and PKCε and PKCα (Qiagen; Hilden, Germany). For transfection of GPCRs, 5 μg of cDNA for the human EP1 receptor (Missouri S&T cDNA Resource Center; Rolla, MO) or human EP3, CysLT1, and BLT1 receptors (Origene; Rockville, MD) was introduced into QBI293 cells stably expression hAPP_695_ using Lipofectamine 2000 (Life Technologies; Grand Island, NY) in 70% confluent 10 cm dishes. Cells were replated into 6-well culture plates and treated as indicated after adhering to the plates. Primary hippocampal neurons from embryonic Sprague Dawley rats or cortical neurons from embryonic CD1 mice were obtained from a core facility (University of Pennsylvania; Philadelphia, PA). The neurons were cultured in 1xNeurobasal A medium with B27 and penicillin/streptomycin (Life Technologies, Grand Island, NY). Mouse neuron medium was also supplemented with 1xGlutamax (Life Technologies, Grand Island, NY). All neuronal cultures had 2.3 μM cytosine arabinoside (Sigma-Aldrich, St. Louis, MO) added starting 2 days after plating to inhibit the proliferation of mitotic cells (largely astrocytes), and the cultures were allowed to grow 14 – 16 days prior to treatment. QBI293 cells or neurons were treated as indicated in the figure legends with IBOP, LTD4, LTB4 (Cayman Chemical Company, Inc.; Ann Arbor, MI); 17PGE2 (Santa Cruz, Biotechnologies, Inc.; Dallas, TX); and/or PMA, ionomycin, Y-27632, and Gο6983 (Sigma-Aldrich; St. Louis, MO).

### RNA isolation and qRT-PCR

Cells were washed, lysed, and RNA was isolated according to the RNEasy manufacturer’s protocol (Qiagen; Hilden, Germany). RNA (3 μg) was converted to cDNA using SuperScript III Reverse Transcriptase (Life Technologies; Grand Island, NY). For RT-PCR, the cDNA was diluted 1:8 in water and 7.6 μL of the product was added to each well containing SYBR Green Master Mix (Life Technologies; Grand Island, NY) and forward and reverse primers at a final concentration that was determined to yield the greatest amplification efficiency (100 nM for APP and 300 nM for all other primer sets). qRT-PCR was run on the Applied Biosystems 7500 Fast Real-time PCR system (Life Technologies; Grand Island, NY) using the ΔΔC_t_ comparative method of the analyzed gene product vs. GAPDH. The sequences of the primers used for qRT-PCR analysis are shown in Table 1.

### Immunoblot analysis

Cells were lysed in RIPA buffer (50 mM Tris, 150 mM NaCl, 0.5% sodium deoxycholate, 0.1% SDS, 1% NP-40, 5 mM EDTA, pH 8.0) containing a protease inhibitor cocktail (Sigma-Aldrich; St. Louis, MO) and 2 mM PMSF. Lysate was vortexed and then centrifuged at 13,000 x *g* at 4 °C for 30 min. For examination of APP levels in brains of 5XFAD transgenic mice, cortex and hippocampus were removed from freshly dissected brains obtained from mice euthanized according to protocols approved by the Institutional Animal Care and Use Committee (IACUC). The samples were quick frozen on dry ice, and subsequently homogenized and sonicated in 2% SDS containing the protease inhibitor cocktail and 2 mM PMSF. The homogenates were centrifuged at 40,000 x *g* for 30 min at room temperature. The supernatants were collected and the pellets were again sonicated in 2% SDS containing protease inhibitors and 2 mM PMSF. After another centrifugation at 40,000 x *g* for 30 min at room temperature, the supernatant was combined with the initial supernatant. The total protein concentration of cell- or brain-derived samples was determined by BCA assay (Pierce Biotechnologies; Rockford, IL). For most analyses, proteins were separated on 10% SDS-PAGE gels, transferred to nitrocellulose membranes, blocked in blocking buffer (LiCor Biosciences; Lincoln, NE), and incubated overnight with one of the following primary antibodies: APP (amino-terminal, 22C11^68^ and Karen^69^; carboxyl-terminal, 5685^31^); α-tubulin (Covance; Princeton, NJ); GAPDH (Millipore; Billerica, MA); Gα_q_, Gα_12_, PKCε (Santa Cruz Biotechnologies, Inc.; Dallas, TX); Gα_13_ (AbCam; Cambridge, MA); or PKCα (Cell signaling; Danvers, MA). Membranes were washed 3 times for 10 min in Tris-buffered saline and incubated with IRDye 800CW- or 680RD-conjugated 2^o^ antibodies, followed by imaging and quantification with the Odyssey Imaging System (LI-COR Biosciences; Lincoln, NE). For the analysis of APP and COOH-terminal APP fragments, proteins were separated on 4–12% Bis-Tris gradient gels with MES running buffer (Life Technologies, Grand Island, NY), transferred to nitrocellulose membranes, blocked in 5% milk and incubated overnight with the 5685 APP carboxyl-terminal antibody, followed by treatment with 2^o^ antibodies as above. Membranes of transfers from 10% gels were routinely cut between the 50 kD and 75 kD molecular weight markers prior to primary antibody incubations so as to allow for independent staining of APP species and housekeeping genes. For studies with 5XFAD transgenic mice, the identity of brain samples were masked prior to gel loading so that the individual conducting immunoblot quantification was unaware of the lane assignments.

### Aβ40 and Aβ42 ELISA

Aβ1-40 and Aβ1-42 levels were determined by ELISA as previously described^12^. In 2% SDS cortical homogenates from vehicle- or drug-treated 5XFAD mice, the samples were diluted at least 100-fold, such that the final readings fell within the linear portion of a standard curve.

### COX and 5-LOX Inhibitor Administration to 5XFAD Transgenic Mice

All mouse studies were approved by the University of Pennsylvania IACUC. Male and female 5XFAD transgenic mice^30^ were utilized in these studies, with the age of the mice, the number of mice per group, and their gender distribution listed in the figure legends. The 5XFAD mice express human APP and PS1 that contain 5 mutations associated with familial AD, with germ-line transmission and stable genomic cointegration of both transgenes^30^. Heterozygous male 5XFAD mice (B6/SJL background) were bred with B6/SJL F1 hybrids, and transgenic mice were identified by PCR analysis of tail clips using primers specific for the human APP and human PS1 transgenes. Only mice that were positive for both transgenes were classified as 5XFAD transgenics. Mice from different breedings were grouped together to obtain sufficient group sizes that were within 2 weeks of age. Aged 5XFAD mice were administered either a combination of SC-560 and rofecoxib, a mixture of SC-560, rofecoxib, and licofelone, or vehicle alone for 7 days via drinking water. SC-560 and rofecoxib were formulated at 70 μg/mL each, and licofelone at 0.7 mg/ml, in 3% (v/v) polyethylene glycol 400, 0.5% (w/v) methyl cellulose 400 cP and 10% (w/v) sucrose. The mice were given *ad libitum* access to the water.

### Quantification of TXB2 and PGE2 in Brain Extracts

PGE2 and TXB2 were extracted from mouse brain homogenates and quantified using stabile isotope dilution (SID) LC-MS/MS. Mouse brain hemispheres were homogenized in 10 mM ammonium acetate buffer pH 5.8 (50% w/v) and extracted essentially as described^70^. Briefly, 3 ml of an acetone/saline solution (2:1) with 0.01% butylated hydroxytoluene (BHT) as an antioxidant was added to 0.1 mL (50 mg) of brain homogenate that had been spiked with 1 ng PGE2-d4 and 1 ng TXB2-d4. This mixture was vortexed for 4 min and then centrifuged at 2000 x *g* for 10 min. The supernatant was moved to a new tube and 2 ml of hexane was added. After 1 min of vortex mixing and centrifugation as above, the upper hexane phase was discarded. The lower phase was acidified with 30 μl of 2M acetic acid, followed by the addition of 2 ml of chloroform containing 0.01% BHT. This mixture was vortexed, centrifuged as above, and the lower chloroform phase dried under nitrogen in a 35 °C water bath. Dried samples were reconstituted in 0.1 ml of ethanol for subsequent analysis by LC-MS/MS (Acquity UPLC-TQD; Waters Corporation, Milford, MA, USA). Samples (10 μL) were separated on a BEH C18 column (1.7 um, 2.1 × 50 mm) using a water/acetonitrile gradient with 0.1% formic acid from 5 to 95% acetonitrile over 5 minutes at 0.6 mL/min and 35 °C. Compounds were detected in negative electrospray ionization mode. Source voltages and MS parameters were optimized for PGE2 and TXB2 during direct infusion of standard solutions (Cayman Chemical, Ann Arbor, MI). Analytes and standards were detected using multiple reaction monitoring of their specific collision induced ion transitions as follows (negative *m*/*z*): PGE2 (351 > 271), PGE2-d4 (355 > 275), TXB2 (369 > 169), TXB2-d4 (373 > 173). Standard solutions were prepared with a constant 1 ng of deuterium-labeled internal standard and varying amounts of analyte over concentrations from 1 to 1000 ng/mL. Peak area ratios (analyte/internal standard) were plotted versus standard concentration and a linear regression curve was fit to the data.

### Statistics

Linear mixed-effect models were used to compare the outcomes in cell culture studies when there were three or more treatment groups. The fixed-effects in the linear mixed-effects model were the treatment types and replicate runs, whereas experiment-specific random intercepts were used to account for the correlation between repeated measures within an experiment. Analyses were conducted with SAS v.9.2 (SAS Institute, Inc., Cary, NC). All statistics were two-tailed, with P ≤ 0.05 considered significant. For cell culture or mouse experiments in which only two treatment types were compared, statistical analysis consisted of an unpaired, two-tailed T-test (GraphPad Prism, GraphPad Software, La Jolla, CA). For mouse studies in which three or more treatment conditions were compared, an ANOVA analysis was conducted with either a Dunnett’s or Tukey’s post-hoc multiple comparison test (GraphPad Prism, GraphPad Software, La Jolla, CA). Detailed statistical outcomes are provided in the Supplementary Materials.

## Additional Information

**How to cite this article**: Herbst-Robinson, K. J. *et al.* Inflammatory Eicosanoids Increase Amyloid Precursor Protein Expression via Activation of Multiple Neuronal Receptors. *Sci. Rep.* **5**, 18286; doi: 10.1038/srep18286 (2015).

## Supplementary Material

Supplementary Information

## Figures and Tables

**Table 1:** qPCR primer pairs.

	Sense	Antisense
hGαq	CATCAATGGGTCAGGATACTCTGATGAAG	GTGCATGAGCCTTATTGTGCTCATAC
**hGα12**	CAAGGGCTCAAGGGTTCTTGTTG	CTGATGCCAGAATCCCTCCAGA
**hGα13**	CTGGTGAAGATCCTGCTGCTGG	CCAGCACCCTCATACCTTTGATCAC
**hAPP**	CCAACCAGTGACCATCCAGAACTG	GCACTTGTCAGGAACGAGAAGGG
**hGAPDH**	GAAGGTGAAGGTCGGAGTCAACG	CCAGAGTTAAAAGCAGCCCTGGTG
**hPKCα**	CCACCATTCAAGCCCAAAGTGTG	GGCTGTCCTCGTGTGTGAAGAAC
**hPKCε**	GCTTGAAGCCCACAGCCTG	CTTGTGGCCGTTGACCTGATG
**hPKCβII**	GGATTGGGAGAAACTTGAACGCAAAGAG	CCTGATGACTTCCTGGTCGGG
**hTP**	ACGGAGAAGGAGCTGCTCATC	GCGGCGGAACAGGATATACA
**hEP1**	CCTGTCGGTATCATGGTGGTGTC	GCTTACCGGAAGTGGCTGAGG
**hEP3**	AAGGCCACGGCATCTCAGT	TGATCCCCATAAGCTGAATGG
**hCysLT1**	GAGAAACATGGATGAAACAGGAAATCTGACAG	CAAAGCATAGGTGCTGAGGCG
**hBLT1**	GTCTGCGGAGTCAGCATTGTACG	GTAGCCGACGCCCTATGTCC
