# Supplementary material for: Inflammatory Eicosanoids Increase Amyloid Precursor Protein Expression via Activation of Multiple Neuronal Receptors
Source: Sci Rep. 2015 Dec 17;5:18286. doi: 10.1038/srep18286 (PMC4682150; doi:10.1038/srep18286)
Supplement: Supplementary Information [file srep18286-s1.pdf]

Supplementary Information

Inflammatory Eicosanoids Increase Amyloid Precursor Protein Expression via Activation of Multiple Neuronal Receptors

Herbst-Robinson K.J., Liu L., James M., Yao Y., Xie S., and Brunden K.R.

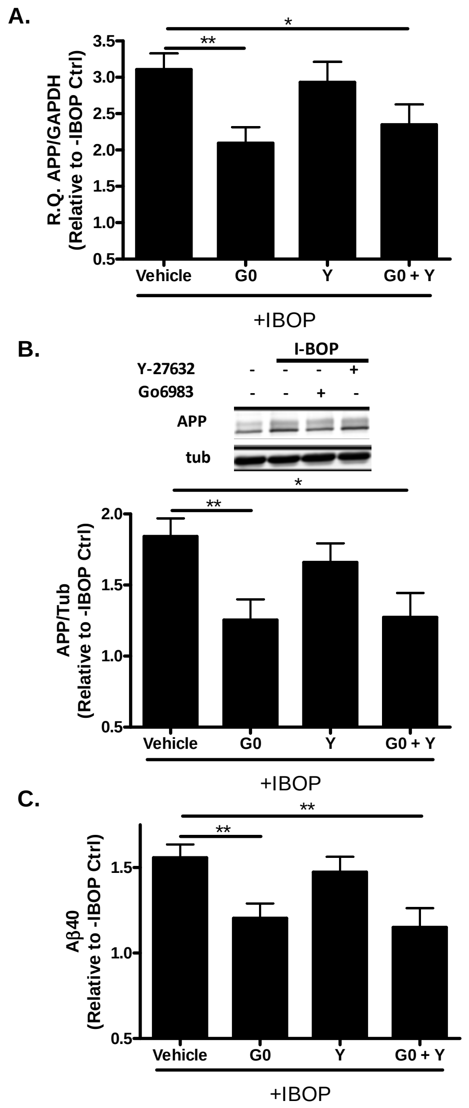

Supplementary Figure 1. PKC inhibition blocks TP receptor-mediated increases in APP and Aβ. (**a-c**) hTP-hAPP cells were pre-incubated with the pan PKC inhibitor, Go6983 (Go; 1 μM) or the ROCK inhibitor, Y-27632 (Y; 10 μM) in the presence of IBOP. (**a**) The IBOP-induced increase in APP mRNA was attenuated in the presence of Go6983, but not Y-27632 [R.Q. values of APP/GAPDH are relative to non-IBOP-treated control cells]. (**b**) The IBOP-induced increase in APP protein expression was attenuated by pretreatment with Go6983, but not Y-27632. A representative blot is included with quantification of APP expression, with values relative to non-IBOP-treated control cells, with normalization to α-tubulin. (**c**) The increase in Aβ1-40 release into medium induced by IBOP treatment was decreased in the presence of Go6983, but not Y-27632 [values are relative to non-IBOP-treated control cells, normalized to total cellular protein content]. Statistical analyses consisted of a mixed-effects model, with values representing estimates from the least squares means fit of the mixed procedure from 2-3 independent studies with 2-5 replicates per study for A. and B., and 3-9 replicates per study for C. Error bars represent SEM; *, $p<0.05$; **, $p<0.01$;

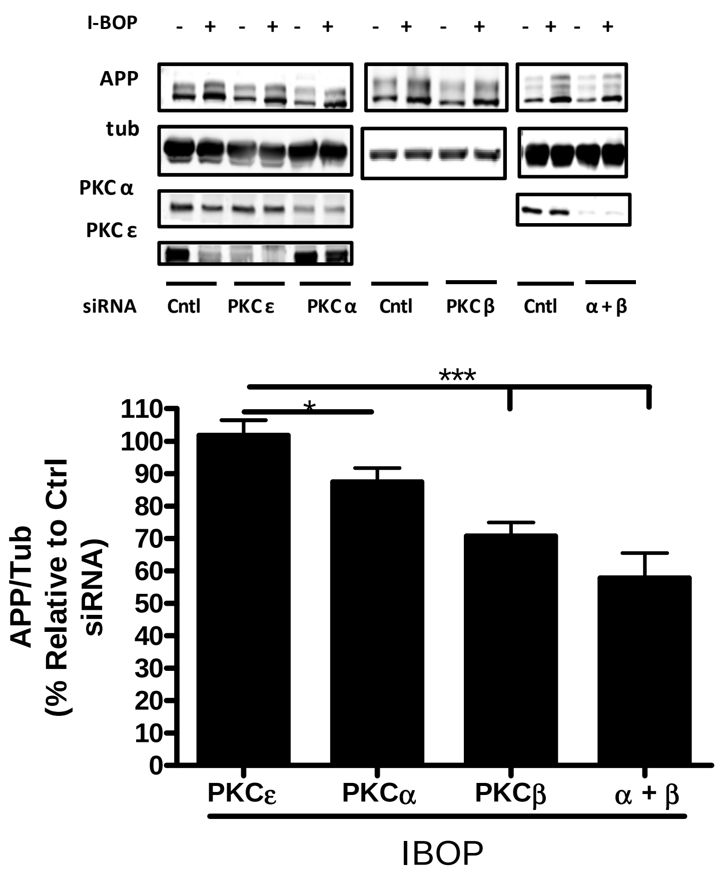

Supplementary Figure 2. Conventional PKC isoforms regulate APP expression. hTP-hAPP cells were transfected with 50 nM of control siRNA or siRNA against PKCε, PKCα, and/or PKCβ and grown for 72 h, with IBOP (10 nM) added over the last 48 h. Only those cells treated with PKCα and/or PKCβ siRNA showed a reduction of IBOP-induced APP protein expression [(top) representative blot and (bottom) quantification of APP with values relative to cells treated with control siRNA and IBOP, with normalization to α-tubulin]. Statistical analyses consisted of a mixed-effects model, with values representing estimates from the least squares means fit of the mixed procedure from two independent studies with 2-3 replicates per study, except for the combined PKCα + PKCβ knockdown, which was conducted in a single experiment with 2 replicate samples. Error bars represent SEM; *, $P<0.05$; ***, $P<0.001$.

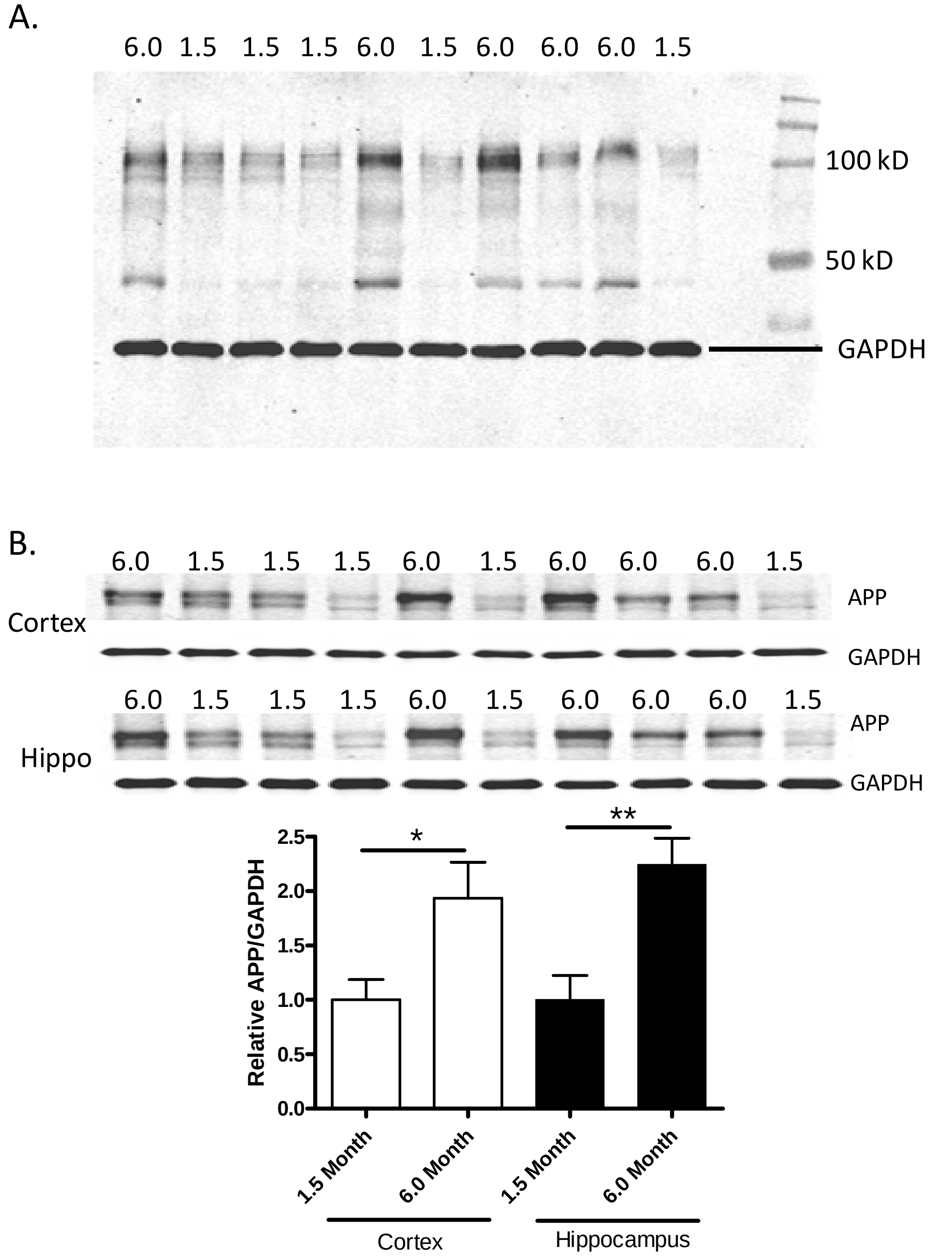

Supplementary Figure 3. Aged 5XFAD mice show increased levels of total APP species. Brain homogenates from 5XFAD mice that were 1.5 or 6.0 months of age (3 males and 2 females/group) underwent immunoblot analysis for determination of APP levels. (**A**) Full 22C11 antibody immunblot image of hippocampal samples, as shown in Figure 6C. Intact APP and sAPP species have an apparent molecular weight of ~95-110 kD[31]. The blot was also probed with a GAPDH antibody. (**B**) A second amino-terminal APP antibody (Karen) also reveals a significant increase of APP species in the cortex and hippocampus of 6.0-month old 5XFAD mice. Lane loading order in the immunoblots are as in Figure 6. Statistical analyses consisted of a two-tailed t-test. Error bars represent SEM ; *, p<0.05; **, p<0.01.

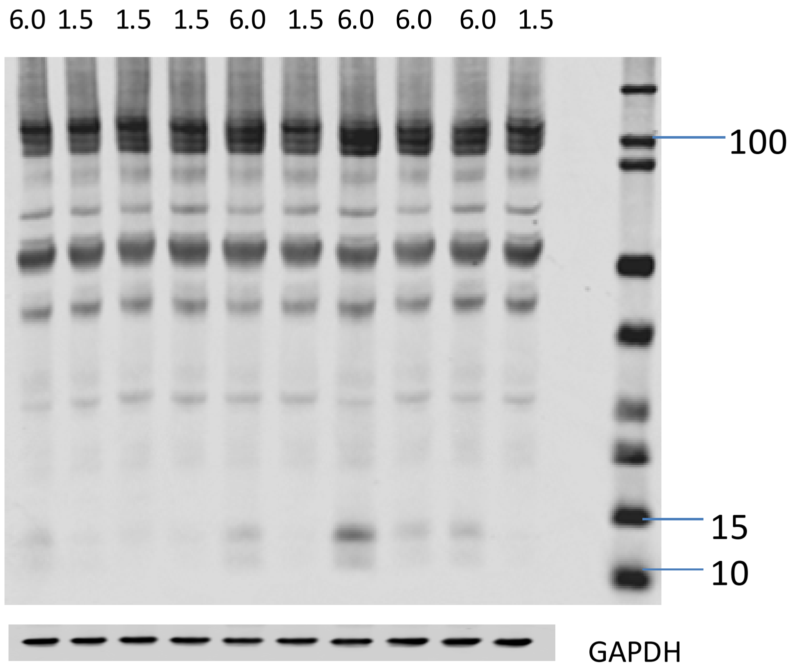

Supplementary Figure 4. Photo of the complete immunblot obtained from cortical homogenates of 1.5-month old and 6.0-month old 5XFAD mice after SDS-PAGE on a 4-12% gradient gel and probing with APP 5685 antibody. Intact APP species (immature and mature) run as multiple species of ~95-110 kD, whereas the APP C99 and C83 COOH-terminal fragments run with apparent molecular weights between 12-14 kD (see [31]). Additional bands correspond to truncated APP and/or non-specific bands that are observed with multiple APP antibodies.

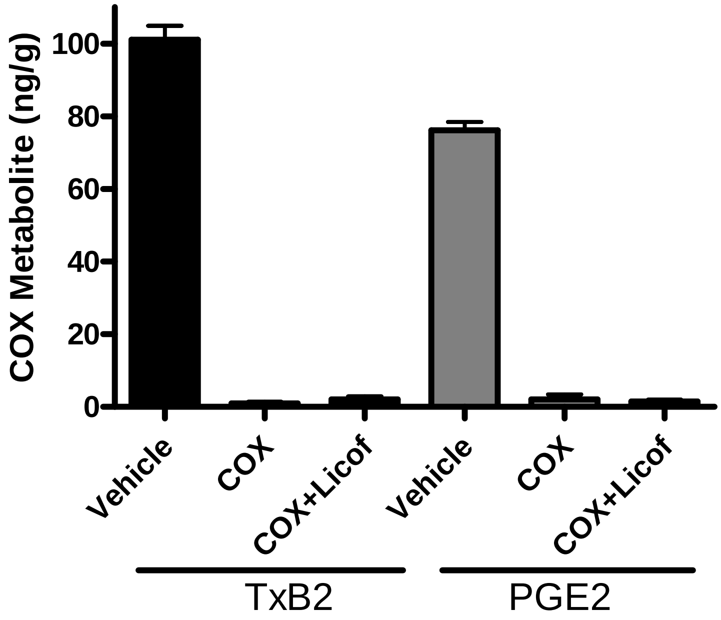

Supplementary Figure 5. Inhibition of TxB2 and PGE2 production in aged 5XFAD mice. The concentration of brain TxB2 and PGE2 were assessed in 5.5-6.0-month old 5XFAD mice after 7 days of oral dosing via drinking water with the combination of SC-560 and rofecoxib (COX) or SC-560 and rofecoxib supplemented with licofelone (COX + Licof). A total of 3-9 mice were analyzed from each treatment group. Error bars represent SEM.

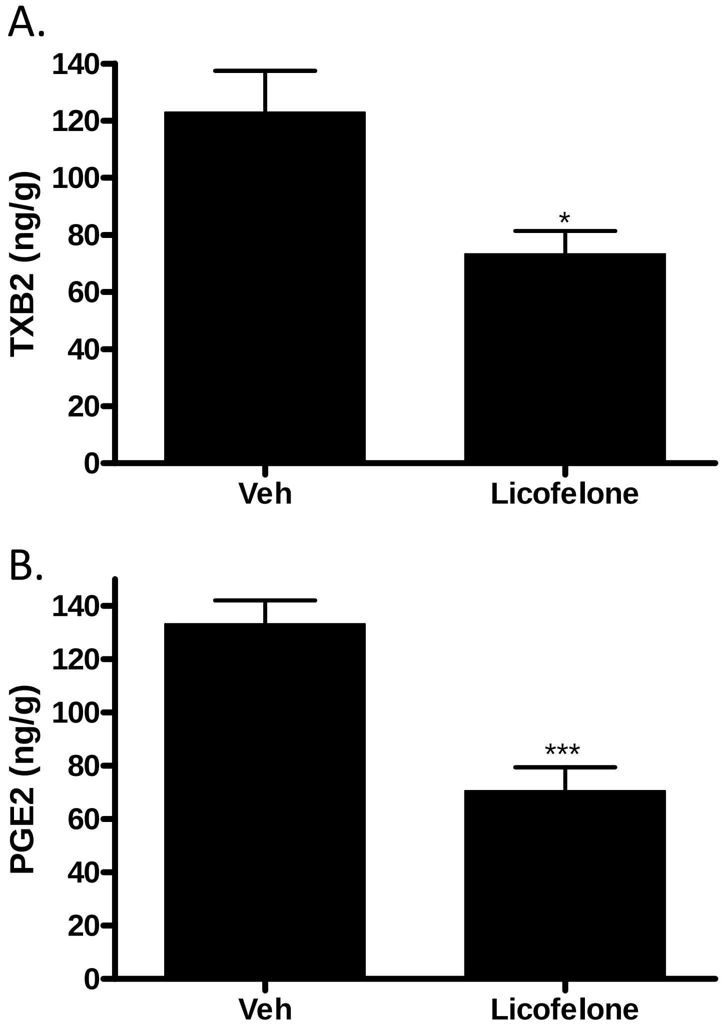

Supplementary Figure 6. Licofelone reduces TxB2 and PGE2 levels in aged 5XFAD mice. 5XFAD mice (5.5-6.0 months of age) were administered 0.7 mg/ml of licofelone suspended in drinking water containing 3% PEG, 0.5% methylcellulose and 10% sucrose, or vehicle alone. After 3 days of administration, the levels of TxB2 (**A**) or PGE2 (**B**) in the cortex were evaluated by LC-MS/MS. Assessment of brain licofelone concentration upon study completion revealed an average exposure of 0.60 +/- 0.30 μM. Statistical analyses consisted of a two-tailed t-test, with n=7 (2 males and 5 females) in each group. Error bars represent SEM; *, $p<0.05$; ***, $p<0.001$.

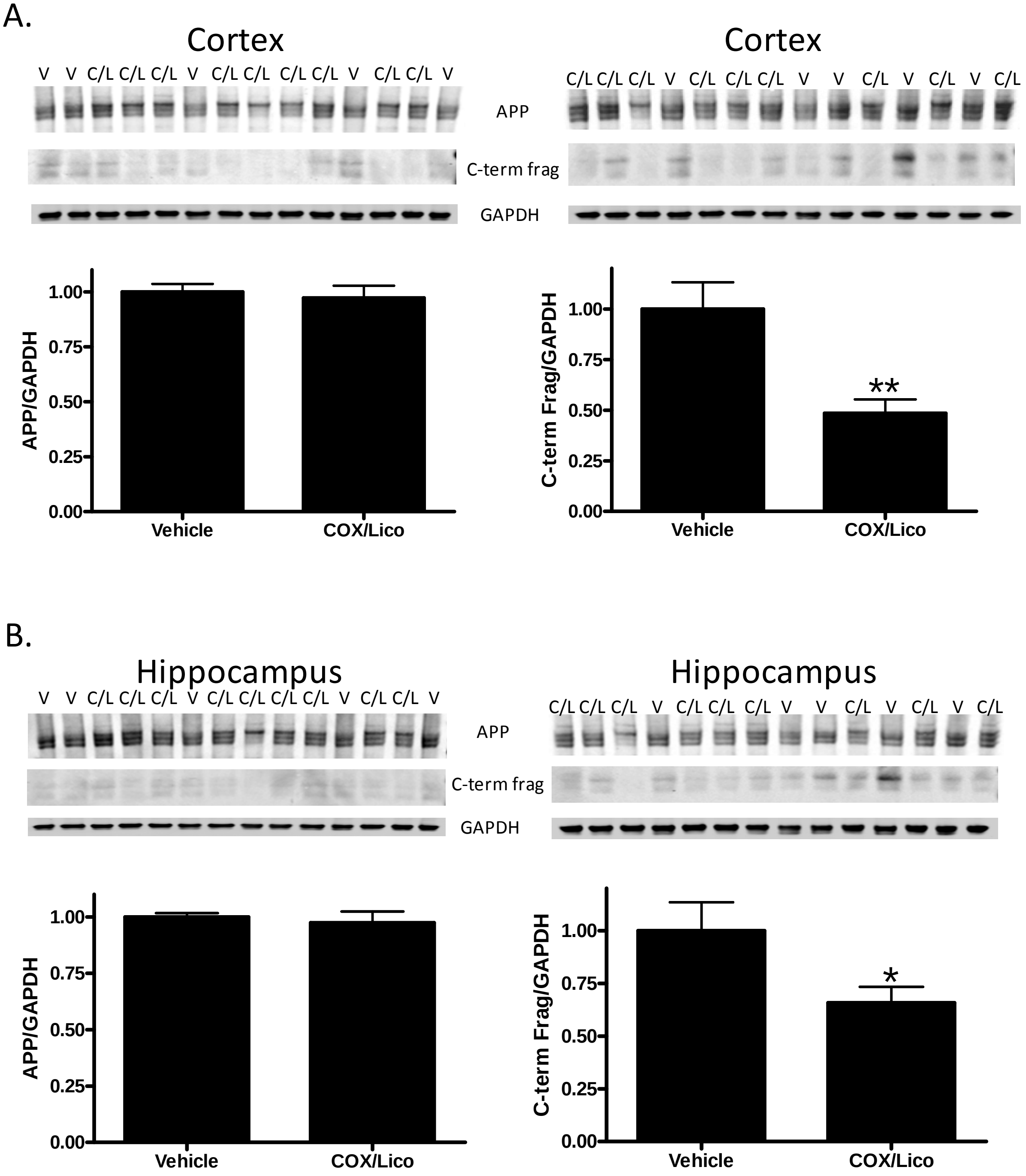

Supplementary Figure 7. COOH-terminal APP antibody (5685) reveals a reduction of COOH-terminal APP fragments, but not intact APP, upon treatment with COX/Licof. Cortical (**A**) and hippocampal (**B**) homogenate samples from vehicle- and COX/Licof-treated aged 5XFAD mice (same samples analyzed with 22C11 antibody in Figure 8) were run on a 4-12% gradient gel and probed with the 5685 antibody. Statistical analyses consisted of a one-way ANOVA with a Tukey's multiple comparison test. Error bars represent SEM ; *,p<0.05; **, p<0.01; n=9-10 per treatment.

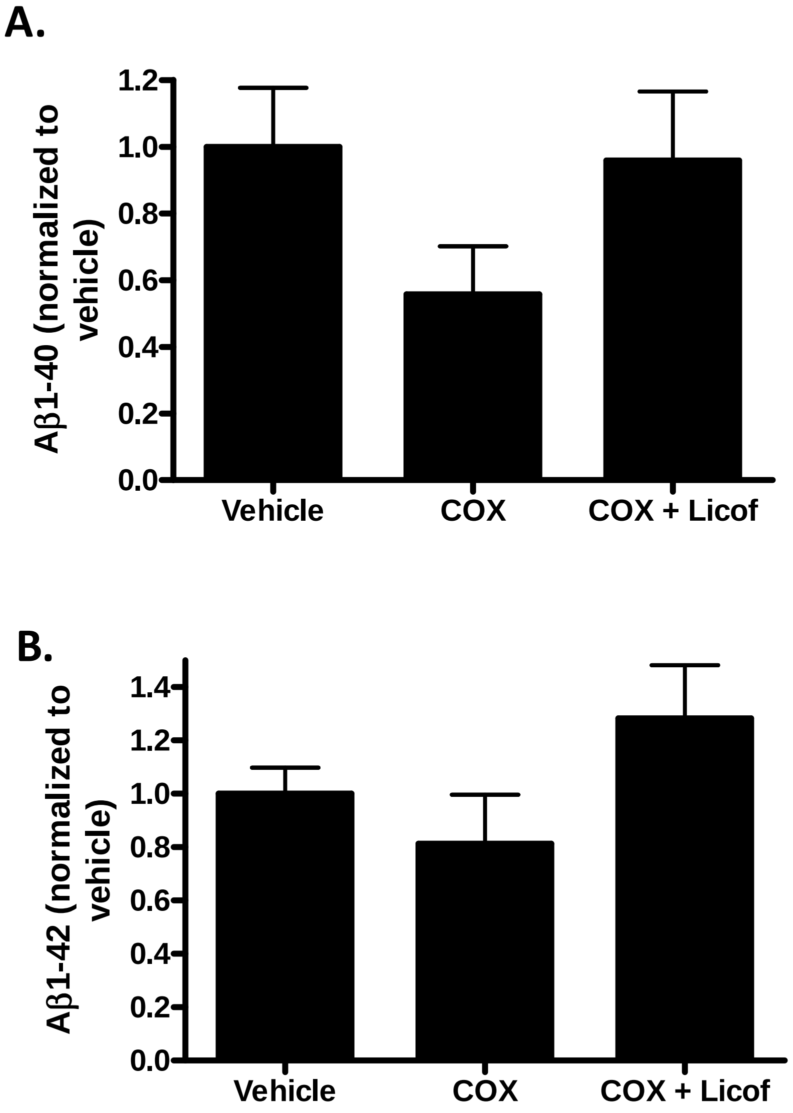

Supplementary Figure 8. Total Aβ levels in drug-treated aged 5XFAD mice. Aβ1-40 (**A**) and Aβ1-42 (**B**) levels were determined by ELISA in 2% SDS cortical brain homogenates prepared from 5.5-6.0 month old 5XFAD mice after 7 days of oral dosing via drinking water with vehicle, the combination of SC-560 and rofecoxib (COX), or SC-560 and rofecoxib supplemented with licofelone (COX + Licof). Brains were from the mice described in Figure 8 (n=9-10/group). Error bars represent SEM.

siRNA	mRNA	Protein
**Gα_q**	97.4 +/- 0.5 %	58.5 +/- 7.4 %
**Gα_{12}**	84.0 +/- 2.0 %	57.7 +/- 23.6 %
**Gα_{13}**	78.6 +/-10.9 %	92.3 +/- 1.3 %

Supplementary Table 1. Knockdown of Gα proteins. hTP-hAPP cells were transfected with 50 nM of control siRNA or siRNA directed against Gαq, Gα_{12}, or Gα_{13} for 24 h prior to subsequent incubation in the absence or presence of IBOP. The extent of Gα mRNA and protein knockdown were determined by qRT-PCR (n=3 replicate samples from a single experiment) and immunoblot analyses (n=4-6 replicate samples from a single experiment). Data are expressed as percent knockdown +/- SD.

siRNA	mRNA	Protein
**PKCε**	N.D.	71.7 +/- 13.3 %
**PKCα**	89.7 +/- 1.3 %	76.1 +/- 10.6 %
**PKCβ**	94.4 +/- 0.6 %	N.D.
**PKCα +<br>PKCβ**	94.4 +/- 1.6 %<br>87.5 +/- 1.1 %	90.1 +/- 2.2 %<br>N.D.

Supplementary Table 2. Knockdown of PKC isoforms. hTP-hAPP cells were transfected with 50 nM of control siRNA or siRNA directed against PKCε, PKCα, and/or PKCβ, and qRT-PCR and immunoblot analyses were performed to determine the efficiency of PKC isoform knockdown after 72 h in culture. Data are from 1-2 independent experiments with 3 replicate samples per experiment, with the results expressed as percent knockdown +/-SD. N. D. = not determined.

Receptor	Mock-transfected Ct	Receptor-transfected Ct
**EP1**	32.7 +/- 0.07	25.3 +/- 0.13
**EP3**	31.1 +/- 0.21	26.7 +/- 0.20
**CysLT1**	>36	20.3 +/- 0.80
**BLT1**	35.4 +/- 0.36	25.1 +/1 0.24

Supplementary Table 3. Expression of eicosanoid receptors in hAPP-expressing QBI293 cells. RNA samples from mock-transfected cells and cells transfected with individual receptor cDNA were subjected to qRT-PCR analysis using receptor-specific primers, with the cycle threshold shown for each population of cells. A lower cycle threshold is indicative of more RNA. The GAPDH cycle threshold values for all samples were within 0.5 cycles of each other, indicative of equal loading of total mRNA in each sample. Three replicate samples from a single transfection of each receptor cDNA were analyzed, and the difference between the mock- and receptor-transfected Ct values were compared.

Treatment	SC-560 (nM)	Rofecoxib (nM)	Licofelone (nM)	Daily Water (ml)	BW -Start (g)	BW - End (g)	BW Change (g)
SC-560 + Rofecoxib	536 +/- 193	419 +/- 204	NA	7.0 +/- 1.3**	32.6 +/- 7.7	31.9 +/- 7.7	-0.7 +/- 1.1
SC-560 + Rofecoxib + Licofelone	225 +/- 61	64 +/- 47	573 +/- 249	2.8 +/- 0.1***, †	26.8 +/- 5.7	25.6 +/- 4.6	-1.2 +/- 1.9
Vehicle	NA	NA	NA	9.6 +/- 2.5	29.2 +/- 4.8	29.0 +/- 5.1	-0.2 +/- 1.1

Supplementary Table 4. The average concentrations and standard deviations of COX and 5-LOX inhibitors in the brains of the 5XFAD mice used to evaluate APP levels after oral dosing via drinking water for 7 days. As the drinking solutions were sweetened with sucrose to reduce compound-induced taste aversion, the vehicle-treated mice consumed more than the ~5 ml of water typically ingested by mice per day. The mice receiving drugs consumed less water per day on average than the vehicle-treated 5XFAD mice, and those receiving the combination of SC-560 + Rofecoxib + licofelone drank significantly less than the SC-560 + Rofecoxib group. However, differences in drinking water consumption among treatment groups did not result in significant differences in body weight (BW) change during the study, and there were not significant differences in group body weights at the start or the end of the study. Statistical analyses consisted of a one-way ANOVA, with a Tukey's multiple comparison test. **, p<0.01; ***, p<0.001 relative to the vehicle group; †, p<0.001 relative to the SC-560 + Rofecoxib group. NA = not applicable.

Supplemental Statistical Information

Figure 1A. Differences of Least Squares Means

Effect	treat	_treat	Estimate	Standard Error	DF	t Value	Pr > |t|	Alpha	Lower	Upper
treat	1:Gq	2:G12	-1.1757	0.3261	11	-3.61	0.0041	0.05	-1.8934	-0.4580
treat	1:Gq	3:G13	-1.4583	0.3059	11	-4.77	0.0006	0.05	-2.1316	-0.7850
treat	1:Gq	4:Control	-1.4134	0.2631	11	-5.37	0.0002	0.05	-1.9926	-0.8342
treat	2:G12	3:G13	-0.2826	0.3325	11	-0.85	0.4134	0.05	-1.0144	0.4491
treat	2:G12	4:Control	-0.2377	0.2936	11	-0.81	0.4353	0.05	-0.8839	0.4085
treat	3:G13	4:Control	0.04492	0.2703	11	0.17	0.8710	0.05	-0.5499	0.6398

Figure 1B. Differences of Least Squares Means

**Effect**	treat	_treat	Estimate	Standard Error	DF	t Value	Pr > |t|	Alpha	Lower	Upper
**treat**	1:Gq	2:G12	-1.0292	0.4122	15	-2.50	0.0247	0.05	-1.9079	-0.1506
**treat**	1:Gq	3:G13	-0.8456	0.4122	15	-2.05	0.0581	0.05	-1.7242	0.03305
**treat**	1:Gq	4:Control	-1.1933	0.3480	15	-3.43	0.0037	0.05	-1.9351	-0.4515
**treat**	2:G12	3:G13	0.1837	0.4389	15	0.42	0.6815	0.05	-0.7518	1.1191
**treat**	2:G12	4:Control	-0.1640	0.3795	15	-0.43	0.6717	0.05	-0.9728	0.6448
**treat**	3:G13	4:Control	-0.3477	0.3795	15	-0.92	0.3740	0.05	-1.1565	0.4611

Figure 2A. Differences of Least Squares Means

Effect	treat	_treat	Estimate	Standard Error	DF	t Value	Pr > |t|	Alpha	Lower	Upper
treat	1:P + G + I	2:P + G0	0.08358	0.2461	9	0.34	0.7419	0.05	-0.4731	0.6402
treat	1:P + G + I	3:P + Iono	-1.2786	0.2503	9	-5.11	0.0006	0.05	-1.8448	-0.7123
treat	1:P + G + I	4:PMA	-0.7712	0.2359	9	-3.27	0.0097	0.05	-1.3048	-0.2375
treat	2:P + G0	3:P + Iono	-1.3621	0.2248	9	-6.06	0.0002	0.05	-1.8707	-0.8536
treat	2:P + G0	4:PMA	-0.8548	0.2087	9	-4.10	0.0027	0.05	-1.3268	-0.3827
treat	3:P + Iono	4:PMA	0.5074	0.2131	9	2.38	0.0412	0.05	0.02530	0.9894

Figure 2B. Differences of Least Squares Means

Effect	treat	_treat	Estimate	Standard Error	DF	t Value	Pr > |t|	Alpha	Lower	Upper
treat	4:PMA	5:PMA + G	0.6377	0.2403	16	2.65	0.0173	0.05	0.1284	1.1470
treat	4:PMA	6:PMA + I	-0.4684	0.1699	16	-2.76	0.0140	0.05	-0.8285	-0.1082
treat	4:PMA	7:P + I + G	0.3279	0.1899	16	1.73	0.1035	0.05	-0.07471	0.7306
treat	5:PMA + G	6:PMA + I	-1.1061	0.2403	16	-4.60	0.0003	0.05	-1.6154	-0.5967
treat	5:PMA + G	7:P + I + G	-0.3098	0.2548	16	-1.22	0.2418	0.05	-0.8500	0.2305
treat	6:PMA + I	7:P + I + G	0.7963	0.1899	16	4.19	0.0007	0.05	0.3936	1.1990

Figure 2C. Differences of Least Squares Means

Effect	treat	_treat	Estimate	Standard Error	DF	t Value	Pr > |t|	Alpha	Lower	Upper
treat	1:P + G + I	4:PMA	-0.6529	0.1524	21	-4.28	0.0003	0.05	-0.9698	-0.3359
treat	1:P + G + I	5:PMA + G	0.2574	0.1603	21	1.61	0.1232	0.05	-0.07591	0.5908
treat	1:P + G + I	6:PMA + I	-0.6151	0.1803	21	-3.41	0.0026	0.05	-0.9901	-0.2401
treat	4:PMA	5:PMA + G	0.9103	0.1284	21	7.09	<.0001	0.05	0.6433	1.1773
treat	4:PMA	6:PMA + I	0.03772	0.1624	21	0.23	0.8185	0.05	-0.2999	0.3754
treat	5:PMA + G	6:PMA + I	-0.8726	0.1679	21	-5.20	<.0001	0.05	-1.2217	-0.5235

**Figure 3A.** Two-Tailed Unpaired T-Test					
		P value	t, df	F-test variance p value	
EP1 Veh vs. 17PGE2		0.0023	6.904, 4	0.261	
EP3 Veh vs. 17PGE2		0.0007	9.418, 4	0.587	
CysLT1 Veh vs. LTD4		0.0025	6.738, 4	0.338	
BLT1 Veh vs. LTB4		0.746	0.347 ,4	0.743	
**Figure 3B.** Two-Tailed Unpaired T-Test					
		P value	t, df	F-test variance p value	
EP1 Veh vs. 17PGE2		0.0198	3.760, 4	0.106	
EP3 Veh vs. 17PGE2		0.0097	4.645, 4	0.799	
CysLT1 Veh vs. LTD4		0.1024	2.111, 4	0.444	
BLT1 Veh vs. LTB4		0.7027	0.4101, 4	0.795	
**Figure 3C.** Two-Tailed Unpaired T-Test and One-Sample T-Test					
		P value	t, df	F-test variance p value	
EP1 Veh vs. 17PGE2		0.004	40420, 16	0.671	
EP3 Veh vs. 17PGE2		<0.0001	10.74, 15	0.629	
CysLT1 Veh vs. LTD4		<0.0001	17.117, 8	One-sample t-test	

**Figure 4A.** Differences of Least Squares Means										
Effect	treat	_treat	Estimate	Standard Error	DF	t Value	Pr > |t|	Alpha	Lower	Upper
treat	1:IBOP	2:IBOP+51280	0.5660	0.1293	24	4.38	0.0002	0.05	0.2991	0.8330
treat	1:IBOP	8:Control	0.7590	0.1293	24	5.87	<.0001	0.05	0.4920	1.0259
treat	2:IBOP+51280	8:Control	0.1930	0.1293	24	1.49	0.1487	0.05	-0.07398	0.4599
**Figure 4B.** Differences of Least Squares Means										
Effect	treat	_treat	Estimate	Standard Error	DF	t Value	Pr > |t|	Alpha	Lower	Upper
treat	3:LTD4	4:LTD4 +Pran	0.3520	0.1027	24	3.43	0.0022	0.05	0.1400	0.5640
treat	3:LTD4	8:Control	0.5123	0.1027	24	4.99	<.0001	0.05	0.3003	0.7243
treat	4:LTD4 +Pran	8:Control	0.1603	0.1027	24	1.56	0.1317	0.05	-0.05170	0.3723
**Figure 4C.** Differences of Least Squares Means										
**Effect**	treat	_treat	Estimate	Standard Error	DF	t Value	Pr > |t|	Alpha	Lower	Upper
**treat**	5:PGE	6:PGE + ONO	-0.05214	0.1355	24	-0.38	0.7038	0.05	-0.3319	0.2276
**treat**	5:PGE	8:Control	0.5650	0.1355	24	4.17	0.0003	0.05	0.2853	0.8447
**treat**	6:PGE + ONO	8:Control	0.6171	0.1355	24	4.55	0.0001	0.05	0.3374	0.8968
**Figure 4D.** Differences of Least Squares Means										
Effect	treat	_treat	Estimate	Standard Error	DF	t Value	Pr > |t|	Alpha	Lower	Upper
treat	5:PGE	7:PGE + L	0.5337	0.1723	24	3.10	0.0049	0.05	0.1781	0.8894
treat	5:PGE	8:Control	0.7174	0.1723	24	4.16	0.0003	0.05	0.3617	1.0730
treat	7:PGE + L	8:Control	0.1836	0.1723	24	1.07	0.2972	0.05	-0.1720	0.5392

**Figure 5A.** Differences of Least Squares Means										
Effect	treat	_treat	Estimate	Standard Error	DF	t Value	Pr > |t|	Alpha	Lower	Upper
treat	1:IBOP	2:LTD	0.06221	0.1588	25	0.39	0.6986	0.05	-0.2649	0.3893
treat	1:IBOP	3:PGE	-0.1067	0.1588	25	-0.67	0.5079	0.05	-0.4338	0.2204
treat	1:IBOP	4:I+L+P	-0.05548	0.1588	25	-0.35	0.7297	0.05	-0.3825	0.2716
treat	1:IBOP	9:Control	0.7321	0.1700	25	4.31	0.0002	0.05	0.3819	1.0823
treat	2:LTD	3:PGE	-0.1689	0.1588	25	-1.06	0.2977	0.05	-0.4960	0.1582
treat	2:LTD	4:I+L+P	-0.1177	0.1588	25	-0.74	0.4655	0.05	-0.4448	0.2094
treat	2:LTD	9:Control	0.6699	0.1700	25	3.94	0.0006	0.05	0.3197	1.0201
treat	3:PGE	4:I +L+P	0.05121	0.1588	25	0.32	0.7498	0.05	-0.2759	0.3783
treat	3:PGE	9:Control	0.8388	0.1700	25	4.93	<.0001	0.05	0.4886	1.1890
treat	4:I+L+P	9:Control	0.7876	0.1700	25	4.63	<.0001	0.05	0.4374	1.1378

**Figure 5B.** Differences of Least Squares Means										
Effect	treat	_treat	Estimate	Standard Error	DF	t Value	Pr > |t|	Alpha	Lower	Upper
treat	1:IBOP	2:LTD	-0.03695	0.3678	25	-0.10	0.9208	0.05	-0.7944	0.7205
treat	1:IBOP	3:PGE	-0.3674	0.3678	25	-1.00	0.3274	0.05	-1.1249	0.3901
treat	1:IBOP	9:Control	0.8903	0.3737	25	2.38	0.0251	0.05	0.1206	1.6599
treat	1:IBOP	I+L+P	0.05848	0.3678	25	0.16	0.8750	0.05	-0.6990	0.8159
treat	2:LTD	3:PGE	-0.3304	0.3678	25	-0.90	0.3775	0.05	-1.0879	0.4270
treat	2:LTD	9:Control	0.9272	0.3737	25	2.48	0.0202	0.05	0.1575	1.6969
treat	2:LTD	I+L+P	0.09542	0.3678	25	0.26	0.7974	0.05	-0.6620	0.8529
treat	3:PGE	9:Control	1.2577	0.3737	25	3.37	0.0025	0.05	0.4880	2.0273
treat	3:PGE	I+L+P	0.4259	0.3678	25	1.16	0.2578	0.05	-0.3316	1.1833
treat	9:Control	I+L+P	-0.8318	0.3737	25	-2.23	0.0353	0.05	-1.6015	-0.06210

**Figure 5C.** Differences of Least Squares Means										
Effect	treat	_treat	Estimate	Standard Error	DF	t Value	Pr > |t|	Alpha	Lower	Upper
treat	5:I Lo	6:P Lo	0.1711	0.2394	38	0.71	0.4793	0.05	-0.3136	0.6557
treat	5:I Lo	7:I Hi	-0.9561	0.2631	38	-3.63	0.0008	0.05	-1.4887	-0.4234
treat	5:I Lo	8:I Lo + P Lo	-0.8797	0.2704	38	-3.25	0.0024	0.05	-1.4271	-0.3324
treat	5:I Lo	9:Control	0.9780	0.2708	38	3.61	0.0009	0.05	0.4298	1.5261
treat	6:P Lo	7:I Hi	-1.1271	0.2390	38	-4.72	<.0001	0.05	-1.6109	-0.6433
treat	6:P Lo	8:I Lo + P Lo	-1.0508	0.2650	38	-3.97	0.0003	0.05	-1.5872	-0.5144
treat	6:P Lo	9:Control	0.8069	0.2916	38	2.77	0.0087	0.05	0.2166	1.3972
treat	7:I Hi	8:I Lo + P Lo	0.07633	0.2409	38	0.32	0.7531	0.05	-0.4113	0.5639
treat	7:I Hi	9:Control	1.9340	0.2959	38	6.54	<.0001	0.05	1.3349	2.5331
treat	8:I Lo + P Lo	9:Control	1.8577	0.2992	38	6.21	<.0001	0.05	1.2519	2.4634

**Figure 6A.** One-way analysis of variance				
P value	P<0.0001			
P value summary	***			
Are means signif. different? (P < 0.05)	Yes			
Number of groups	4			
F	22.54			
R squared	0.8601			
ANOVA Table	SS	df	MS	
Treatment (between columns)	1.574	3	0.5248	
Residual (within columns)	0.2561	11	0.02328	
Total	1.83	14		
Tukey's Multiple Comparison Test	Mean Diff.	q	P value	95% CI of diff
WT 1.5 vs Tg 1.5	0.1628	1.975	P > 0.05	-0.1879 to 0.5135
WT 1.5 vs WT 6.5	-0.1473	1.788	P > 0.05	-0.4980 to 0.2034
WT 1.5 vs Tg 6.5	-0.6807	8.261	P < 0.001	-1.031 to -0.3300
Tg 1.5 vs WT 6.5	-0.3101	4.065	P > 0.05	-0.6347 to 0.01461
Tg 1.5 vs Tg 6.5	-0.8435	11.06	P < 0.001	-1.168 to -0.5188
WT 6.5 vs Tg 6.5	-0.5334	6.993	P < 0.01	-0.8581 to -0.2088

**Figure 6B.** One-way analysis of variance				
P value	0.0019			
P value summary	**			
Are means signif. different? (P < 0.05)	Yes			
Number of groups	4			
F	10.64			
R squared	0.7615			
ANOVA Table	SS	df	MS	
Treatment (between columns)	1.994	3	0.6646	
Residual (within columns)	0.6245	10	0.06245	
Total	2.618	13		
Tukey's Multiple Comparison Test	Mean Diff.	q	P value	95% CI of diff
WT 1.5 vs Tg 1.5	-0.1256	0.8708	P > 0.05	-0.7499 to 0.4986
WT 1.5 vs WT 6.5	0.2013	1.492	P > 0.05	-0.3827 to 0.7853
WT 1.5 vs Tg 6.5	-0.7515	5.568	P < 0.05	-1.335 to -0.1675
Tg 1.5 vs WT 6.5	0.3269	2.422	P > 0.05	-0.2570 to 0.9109
Tg 1.5 vs Tg 6.5	-0.6258	4.637	P < 0.05	-1.210 to -0.04187
WT 6.5 vs Tg 6.5	-0.9528	7.625	P < 0.01	-1.493 to -0.4121

**Figure 6C.** Two-Tailed Unpaired T-Test			
	P value	t, df	F-test variance p value
Cortex 1.5 M vs. 6.0 M	0.0329	2.575, 8	0.131
Hippo 1.5 M vs. 6.0 M	0.0431	2.401, 8	0.134

**Figure 7A**	Two-Tailed Unpaired T-Test				
		P value	t, df	F-test variance p value	
APP 1.5M vs 6.0M		0.9334	0.0862, 8	0.128	
COOH-APP 1.5M vs 6.0M		0.0348	2.539, 8	<0.0001	
**Figure 7B**	Two-Tailed Unpaired T-Test				
		P value	t, df	F-test variance p value	
APP 1.5M vs 6.0M		0.2908	1.131, 8	0.791	
COOH-APP 1.5M vs 6.0M		0.0013	4.805, 8	0.004	

**Figure 8A.** One-way analysis of variance					
P value	P<0.0001				
P value summary	***				
Are means signif. different? (P < 0.05)	Yes				
Number of groups	3				
F	14				
R squared	0.4445				
Bartlett's test for equal variances					
Bartlett's statistic (corrected)	5.573				
P value	0.0616				
P value summary	ns				
Do the variances differ signif. (P < 0.05)	No				
ANOVA Table	SS	df	MS		
Treatment (between columns)	1.2	2	0.6001		
Residual (within columns)	1.5	35	0.04286		
Total	2.7	37			
Tukey's Multiple Comparison Test	Mean Diff.	q	P value	95% CI of diff	
Vehicle vs COX	0.3048	5.188	P < 0.01	0.1013 to 0.5084	
Vehicle vs COX/Licof	0.3959	6.737	P < 0.001	0.1923 to 0.5994	
COX vs COX/Licof	0.09102	1.319	P > 0.05	-0.1480 to 0.3301	
**Figure 8B.** One-way analysis of variance					
P value	P<0.0001				
P value summary	***				
Are means signif. different? (P < 0.05)	Yes				
Number of groups	3				
F	13.04				
R squared	0.427				
Bartlett's test for equal variances					
Bartlett's statistic (corrected)	9.381				
P value	0.0092				
P value summary	**				
Do the variances differ signif. (P < 0.05)	Yes				
ANOVA Table	SS	df	MS		
Treatment (between columns)	1.474	2	0.7369		
Residual (within columns)	1.978	35	0.05651		
Total	3.452	37			
Tukey's Multiple Comparison Test	Mean Diff.	q	P value	95% CI of diff	
Vehicle vs COX	0.3501	5.19	P < 0.01	0.1164 to 0.5839	
Vehicle vs COX/Licof	0.4308	6.386	P < 0.001	0.1971 to 0.6645	
COX vs COX/Licof	0.08069	1.018	P > 0.05	-0.1938 to 0.3552	

**Supplemental Figure 1A.** Differences of Least Squares Means										
Effect	treat	_treat	Estimate	Standard Error	DF	t Value	Pr > |t|	Alpha	Lower	Upper
treat	1:G0	2:Y	-0.8378	0.3461	16	-2.42	0.0277	0.05	-1.5714	-0.1042
treat	1:G0	3:G + Y	-0.2531	0.3461	16	-0.73	0.4750	0.05	-0.9868	0.4805
treat	1:G0	4:Vehicle	-1.0136	0.3038	16	-3.34	0.0042	0.05	-1.6576	-0.3695
treat	2:Y	3:G + Y	0.5847	0.3815	16	1.53	0.1449	0.05	-0.2241	1.3934
treat	2:Y	4:Vehicle	-0.1758	0.3461	16	-0.51	0.6185	0.05	-0.9094	0.5579
treat	3:G + Y	4:Vehicle	-0.7604	0.3461	16	-2.20	0.0431	0.05	-1.4940	-0.02679

**Supplemental Figure 1B.** Differences of Least Squares Means										
Effect	treat	_treat	Estimate	Standard Error	DF	t Value	Pr > |t|	Alpha	Lower	Upper
treat	1:G0	2:Y	-0.4057	0.1966	16	-2.06	0.0556	0.05	-0.8223	0.01103
treat	1:G0	3:G + Y	-0.02015	0.2217	16	-0.09	0.9287	0.05	-0.4901	0.4498
treat	1:G0	4:Vehicle	-0.5893	0.1932	16	-3.05	0.0076	0.05	-0.9989	-0.1797
treat	2:Y	3:G + Y	0.3855	0.2158	16	1.79	0.0930	0.05	-0.07193	0.8429
treat	2:Y	4:Vehicle	-0.1837	0.1843	16	-1.00	0.3339	0.05	-0.5744	0.2071
treat	3:G + Y	4:Vehicle	-0.5692	0.2121	16	-2.68	0.0163	0.05	-1.0188	-0.1195

**Supplemental Figure 1C.** Differences of Least Squares Means										
Effect	treat	_treat	Estimate	Standard Error	DF	t Value	Pr > |t|	Alpha	Lower	Upper
treat	1:G0	2:Y	-0.2682	0.1174	43	-2.28	0.0273	0.05	-0.5050	-0.03144
treat	1:G0	3:G + Y	0.05302	0.1345	43	0.39	0.6953	0.05	-0.2182	0.3242
treat	1:G0	4:Vehicle	-0.3535	0.1140	43	-3.10	0.0034	0.05	-0.5835	-0.1236
treat	2:Y	3:G + Y	0.3213	0.1354	43	2.37	0.0222	0.05	0.04812	0.5944
treat	2:Y	4:Vehicle	-0.08531	0.1174	43	-0.73	0.4715	0.05	-0.3221	0.1515
treat	3:G + Y	4:Vehicle	-0.4066	0.1345	43	-3.02	0.0042	0.05	-0.6777	-0.1354

**Supplemental Figure 2.** Differences of Least Squares Means										
Effect	treat	_treat	Estimate	Standard Error	DF	t Value	Pr > |t|	Alpha	Lower	Upper
treat	1:E	2:A	14.3694	6.3621	10	2.26	0.0475	0.05	0.1937	28.5451
treat	1:E	3:B	31.0915	6.3621	10	4.89	0.0006	0.05	16.9158	45.2672
treat	1:E	4:A + B	43.9483	8.8014	10	4.99	0.0005	0.05	24.3376	63.5590
treat	2:A	3:B	16.7221	6.0241	10	2.78	0.0196	0.05	3.2996	30.1446
treat	2:A	4:A + B	29.5789	8.7217	10	3.39	0.0069	0.05	10.1457	49.0121
treat	3:B	4:A + B	12.8568	8.7217	10	1.47	0.1712	0.05	-6.5764	32.2900

**Supplemental Figure 3B.** Two-Tailed Unpaired T-Test					
		P value	t, df	F-test variance p value	
Cortex 1.5M vs 6.0M		0.0395	2.456, 8	0.293	
Hippo 1.5M vs 6.0M		0.0056	3.747, 8	0.879	

**Supplemental Figure 6A.** Two-Tailed Unpaired T-Test					
		P value	t, df	F-test variance p value	
Vehicle vs Licof		0.0116	2.975, 12	0.189	
**Supplemental Figure 6B.** Two-Tailed Unpaired T-Test					
		P value	t, df	F-test variance p value	
Vehicle vs Licof		0.0003	4.965, 12	0.997	

**Supplemental Figure 7A.** Two-Tailed Unpaired T-Test				
	P value	t, df	F-test variance p value	
APP Vehicle vs COX/Licof	0.6747	0.4270, 17	0.266	
COOH-APP Vehicle vs COX/Licof	0.0038	3.355, 17	0.059	
**Supplemental Figure 7B.** Two-Tailed Unpaired T-Test				
	P value	t, df	F-test variance p value	
APP Vehicle vs COX/Licof	0.6167	0.5098, 17	0.005	
COOH-APP Vehicle vs COX/Licof	0.0468	2.144, 17	0.096	

**Supplemental Figure 8A.** One-way analysis of variance					
P value	0.1765				
P value summary	ns				
Are means signif. different? (P < 0.05)	No				
Number of groups	3				
F	1.86				
R squared	0.1296				
Bartlett's test for equal variances					
Bartlett's statistic (corrected)	1				
P value	0.6065				
P value summary	ns				
Do the variances differ signif. (P < 0.05)	No				
ANOVA Table	SS	df	MS		
Treatment (between columns)	1.098	2	0.5492		
Residual (within columns)	7.38	25	0.2952		
Total	8.478	27			
Dunnett's Multiple Comparison Test	Mean Diff.	q	P value	95% CI of diff	
Vehicle vs COX	0.4422	1.771	P > 0.05	-0.1432 to 1.028	
Vehicle vs COX + Licof	0.0416	0.1666	P > 0.05	-0.5438 to 0.6270	

**Supplemental Figure 8B.** One-way analysis of variance					
P value	0.1476				
P value summary	ns				
Are means signif. different? (P < 0.05)	No				
Number of groups	3				
F	2.067				
R squared	0.1419				
Bartlett's test for equal variances					
Bartlett's statistic (corrected)	3.581				
P value	0.1669				
P value summary	ns				
Do the variances differ signif. (P < 0.05)	No				
ANOVA Table	SS	df	MS		
Treatment (between columns)	1.008	2	0.5042		
Residual (within columns)	6.098	25	0.2439		
Total	7.106	27			
Tukey's Multiple Comparison Test	Mean Diff.	q	P value	95% CI of diff	
Vehicle vs COX	0.1864	1.162	P > 0.05	-0.3791 to 0.7519	
Vehicle vs COX + Licof	-0.2834	1.766	P > 0.05	-0.8489 to 0.2821	
COX vs COX + Licof	-0.4698	2.854	P > 0.05	-1.050 to 0.1104	

**Supplemental Table 4 (Drinking Water).** One-way analysis of variance					
P value	P<0.0001				
P value summary	***				
Are means signif. different? (P < 0.05)	Yes				
Number of groups	3				
F	39.55				
R squared	0.7526				
Bartlett's test for equal variances					
Bartlett's statistic (corrected)	39.04				
P value	P<0.0001				
P value summary	***				
Do the variances differ signif. (P < 0.05)	Yes				
ANOVA Table	SS	df	MS		
Treatment (between columns)	227.3	2	113.7		
Residual (within columns)	74.71	26	2.874		
Total	302	28			
Tukey's Multiple Comparison Test	Mean Diff.	q	P value	95% CI of diff	
Vehicle vs COX	2.53	4.697	P < 0.01	0.6357 to 4.425	
Vehicle vs COX/LOX	6.753	12.53	P < 0.001	4.858 to 8.647	
COX vs COX/LOX	4.222	7.472	P < 0.001	2.235 to 6.209	

**Supplemental Table 4 (Change in Body Weight).** One-way analysis of variance			
P value	0.2538		
P value summary	ns		
Are means signif. different? (P < 0.05)	No		
Number of groups	3		
F	1.446		
R squared	0.1001		
Bartlett's test for equal variances			
Bartlett's statistic (corrected)	3.604		
P value	0.1649		
P value summary	ns		
Do the variances differ signif. (P < 0.05)	No		
ANOVA Table	SS	df	MS
Treatment (between columns)	5.495	2	2.747
Residual (within columns)	49.39	26	1.900
Total	54.89	28	

**Supplemental Table 4 (Starting Body Weight).** One-way analysis of variance			
P value	0.1418		
P value summary	ns		
Are means signif. different? (P < 0.05)	No		
Number of groups	3		
F	2.108		
R squared	0.1395		
Bartlett's test for equal variances			
Bartlett's statistic (corrected)	1.632		
P value	0.4423		
P value summary	ns		
Do the variances differ signif. (P < 0.05)	No		
ANOVA Table	SS	df	MS
Treatment (between columns)	150.3	2	75.17
Residual (within columns)	927.1	26	35.66
Total	1077	28	

**Supplemental Table 4 (Ending Body Weight).** One-way analysis of variance			
P value	0.0923		
P value summary	ns		
Are means signif. different? (P < 0.05)	No		
Number of groups	3		
F	2.615		
R squared	0.1675		
Bartlett's test for equal variances			
Bartlett's statistic (corrected)	2.503		
P value	0.2861		
P value summary	ns		
Do the variances differ signif. (P < 0.05)	No		
ANOVA Table	SS	df	MS
Treatment (between columns)	180.9	2	90.45
Residual (within columns)	899.1	26	34.58
Total	1080	28	
